# Most Important Biomedical and Pharmaceutical Applications of Silicones

**DOI:** 10.3390/ma18112561

**Published:** 2025-05-30

**Authors:** Jerzy J. Chruściel

**Affiliations:** 1 Łukasiewicz Research Network, Lodz Institute of Technology (ŁIT), 19/27 Marii Skłodowskiej-Curie Str., 90-570 Łódź, Poland; jerzy.chrusciel@lit.lukasiewicz.gov.pl; 2 Circular Economy Center (BCG), Environmental Protection Engineering Research Group, Łukasiewicz Research Network, Lodz Institute of Technology (ŁIT), Brzezińska 5/15, 92-103 Łódź, Poland

**Keywords:** silicones, PDMS, siloxanes, silanes, silicone rubbers and gels, POSS, biomedical devices, breast implants, drug delivery, pharmaceutical applications, biocompatibility, physiological properties, antimicrobial properties, toxicity

## Abstract

Many kinds of silicones are a wide family of hybrid inorganic–organic polymers which have valuable physical and chemical properties and find plenty of practical applications, not only industrial, but also numerous medical and pharmaceutical ones, mainly due to their good thermal and chemical stability, hydrophobicity, low surface tension, biocompatibility, and bio-durability. The important biomedical applications of silicones include drains, shunts, and catheters, used for medical treatment and short-term implants; inserts and implants to replace various body parts; treatment, assembly, and coating of various medical devices; breast and aesthetic implants; specialty contact lenses; and components of cosmetics, drugs, and drug delivery systems. The most important achievements concerning the biomedical and pharmaceutical applications of silicones, their copolymers and blends, and also silanes and low-molecular-weight siloxanes have been summarized and updated. The main physiological properties of organosilicon compounds and silicones, and the methods of antimicrobial protection of silicone implants, have also been described and discussed. The toxicity of silicones, the negative effects of breast implants, and the environmental effects of silicone-containing personal care and cosmetic products have been reported and analyzed. Important examples of the 3D printing of silicone elastomers for biomedical applications have been presented as well.

## 1. Introduction

**Figure 1:**
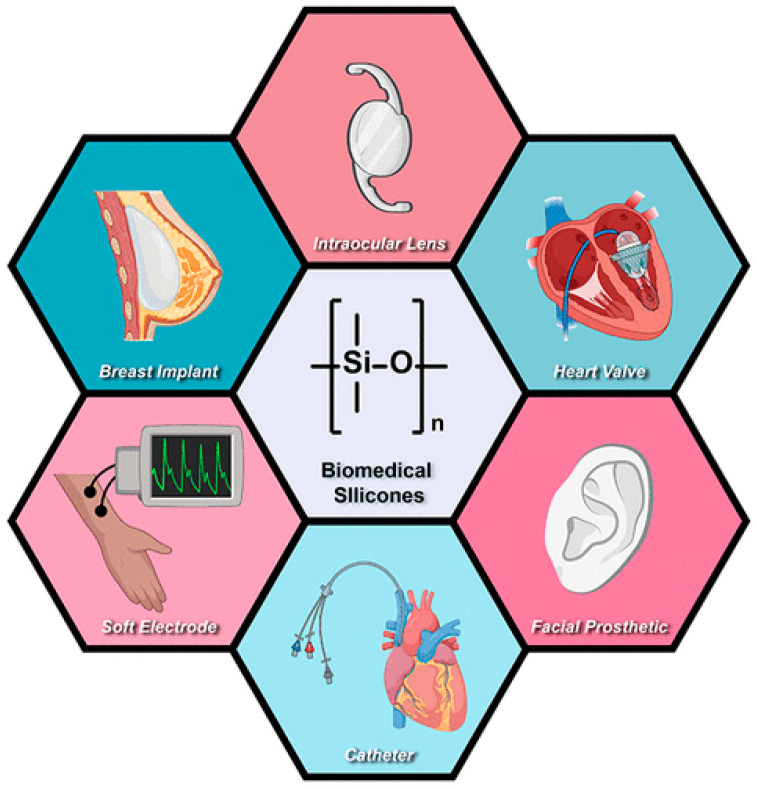
Most important applications of silicones for biomedical devices [5]. Copyright (2023) American Chemical Society. Licensed under CC-BY 4.0.

Silicones are polyorganosiloxanes of different chemical structures, having inorganic silicone–oxygen backbone (≡Si-O-Si≡) and organic substituents. Polydimethylsiloxanes –(Me_2_SiO)_n_– (Me = CH_3_) (PDMS) of linear structures are most often used. Silicones exhibit good thermal and chemical stability. However, the C–Si bonds are sensitive to oxidation due to thermodynamic aspects, but they show kinetic stability. The silicone–oxygen bonds are very strong and the siloxane (Si–O–Si) backbone shows a high degree of flexibility, and high chemical and thermal stability, depending on the kind of organic substituents at silicone atoms [1,2,3,4,5]. Silicone materials have found practical applications in many areas for more than 60 years: from oils used in heating baths to biomedical materials (e.g., as an artificial heart) [6,7]. Improvements in the surface, rheological, mechanical, and electrical properties of silicones were achieved with the use of different additives and led to their newer medical applications, as it was reported by Marmo and Grunlan [4]. An excellent and comprehensive review of numerous application fields of silicone biomaterials was presented by Zare et al. [1]. Many examples of silicone implants in the human body were described in the literature [1,8,9]. The most important examples of applications of biomedical devices fabricated with the use of silicone-based materials are illustrated in Figure 1 [5].

An updated comprehensive review on numerous medical applications of silicone-based biomaterials and devices was published recently. Interactions of silicone implants with soft and bone tissue and various strategies for antimicrobial modifications, including surface treatment and activation with plasma, physiological or chemical alterations, and antibiotic treatment were discussed [10].

### Historical Background of Medical and Pharmaceutical Applications of Silicones

A silicone elastomer tube was implanted for duct repair in a biliary surgery in 1946 [11,12]. Another medical device made of silicones was fabricated in 1950 [13]. Soon, silicones found many other medical applications owing to their valuable properties [14,15]. Silicones have been used in many other life-saving medical devices, for example, as hydrocephalic shunts or pacemakers [16,17]. Silicone tubing is useful in the manufacture of pharmaceuticals and additives in adhesives for transdermal drug delivery systems [18]. Silicones are active pharmaceutical ingredients of antiflatulent and antacid formulations [19,20]. Due to their biocompatibility and different physical forms, as volatile oligomers or high molecular weight polymers, with viscosities from 0.65 cSt to 20 × 10^6^ cSt to viscoelastic compounds and crosslinked elastomers, they are used in medical devices and a variety of pharmaceutical formulations [21,22]. The skin-friendliness and the exceptional biocompatibility of silicones do not cause allergic reactions. Silicones are resistant to low and high temperatures. Moreover, medical devices can also be easily sterilized and are stable for a long time [23]. Silicones of medical grade, and especially PDMS-based elastomers or fluids, fulfill the criteria of the approved standards (e.g., ISO 10993 [24]), including nonirritating and nonsensitizing properties, which are very important for their common use in topical skin and personal care applications. Silicones are widely recognized as biocompatible and show good bio-durability [12]. Due to the backbone flexibility of PDMS materials and low intermolecular interactions of methyl groups at many interfaces, leading to low surface energy and surface tension, they are one of the most favored biocompatible polymers [25].

Due to their exceptional biocompatibility, silicones are one of the most often used materials for the fabrication of medical implants [26,27,28,29]. The excellent chemical stability and elasticity of silicones favor them as an ideal implantation material for long-term applications, e.g., for bile duct repair to various valves and shunts. The silicone ventriculoatrial valve, which was designed by Spitz-Holter in 1956, is still used in an almost unchanged form [27]. Silicones are used in numerous catheters, shunts and drains, contact lenses, orthopedics, and artificial organs, and as components in heart bypass machines, blood oxygenators, kidney dialysis, and many other biomedical devices [27,30]. Other polymeric materials, like natural rubber latex and poly(vinyl chloride) (PVC) previously used for the fabrication of urethral catheters, were almost completely substituted by silicones. The catheters made of silicones are more healthy, although they are more expensive. They significantly reduce the number of catheter insertions, the occurrence of allergic responses, the incidence of phlebitis, the likelihood of mineral encrustations, the potential for bacterial migration, the occurrence of premature balloon deflation, the potential for nosocomial infections, and the frequency of sepsis. Thus, they provide a great comfort for patients [31]. Due to their intrinsic biocompatibility and bio-durability, silicones are one of the most often used groups of biomaterials for the fabrication of catheters. Because of catheter-associated urinary tract infections (CAUTIs), the modification processes of surface properties of silicones are still being continued, with a focus on the surface morphology and roughness and incorporation of special additives, which can prevent the adhesion of bacteria to the silicone catheters [32].

Silicone surfaces were often modified with different polysaccharides: chitosan, carboxymethyl chitosan, hyaluronic acid, fibrinogen, heparin, or cationic aminocellulose. As coating agents for biomedical applications, silicones were often used as polysaccharides. For example, the silicone surface layer modified with 3-(glycidoxypropyl)trimethoxysilane was cured with aminocellulose. Polysaccharides repelled proteins, improved cell viability, and showed antimicrobial properties. Multifunctional coatings prepared with the addition of polysaccharides and antibiotics gave antimicrobial properties. Before grafting polysaccharides, silicone surfaces were biofunctionalized with different antimicrobial reagents. Silicones having a low surface free energy were often activated on their surface in order to increase the interactions of silicones with bioactive agents [26,33,34]. For this purpose, many different chemical oxidations and high-energy etching methods were used: UV/Ozone etching [35,36,37,38], corona discharge [39], plasma treatment [40], and other techniques [34]. The silicone surface was also activated by oxygen and ammonia plasma by Roth et al. [41]. Alternatively, silicone surfaces were first activated by oxygen plasma and next coated with biocompatible polymers [polyethylene glycol (PEG), polyvinylpyrrolidone (PVP)] or 2-hydroxyethyl methacrylate (HEMA) by physicosorption or the preparation of polymer brushes with well-defined nanostructures via the “grafting from” or “grafting to” methods. PDMS surfaces modified with polysaccharides showed long-term hydrophilicity during storage in air. A patterned surface modification of PDMS silicone with cellulose gave spatial alternating hydrophilic and hydrophobic zones on the silicone surface [42].

Maji et al. modified the PDMS silicone rubber surface with a “piranha” and KOH solution [43]. First, the PDMS surface was treated with a “piranha” solution containing hydrogen peroxide and sulphuric acid at a ratio of 2:3. Next, the silicone surface was reacted with a 1M KOH solution. Thus, a hydrophilic silicone surface was obtained and the water contact angle reached 27°, leading to the increased content of hydroxyl groups on the silicone surface [43]. As a result of subsequent treatment, silicone surfaces with new highly reactive functional groups (e.g., hydroxyl groups, carboxyl groups, free oxygen radicals, amino groups, etc.) were formed and the surface free energy of the silicone was increased [35,39,44].

## 2. Biomedical Applications of Silicone Fluids and Thermoplastic Silicone Copolymers

Very thin silicone layers show hydrophobic and antiadhesive (release) properties. Silicone oils have also lubricating properties. The high surface activity of silicone oils enables silicones to find numerous medical, pharmaceutical, and cosmetics applications [45]. Most silicones are physiologically inert (see Section 8). Liquid silicone oils (PDMS) are used for the lubrication of many medical surfaces: needles, syringe pistons, and barrels. They reduce pain during injections [6,12,16,17,18,19,20,21,22,46,47,48,49,50,51,52,53,54,55,56,57]. Such silicone fluids (also called “dimethicones”) with a kinematic viscosity of ~1000 cSt are used in skin protectants, shampoo, creams, and ointments, most likely owing to the high hydrophobicity and spreadability of PDMS. Due to their antifoam properties, dimethicones alone or containing silica (simethicones) are used in gastroenterology in many anitacid and antiflatulent products. They decrease foaming in the stomach without a change in the gastric pH. They act physically and are not metabolized but excreted [58,59].

For biomedical applications, the following properties of silicones are most important [60]:

- Biocompatibility and physiological indifference;
- Excellent oxidation, ultraviolet light, and aging resistance;
- Good elasticity and outstanding dielectric properties at different temperatures.

Different kinds of silanes and silicones were applied for numerous biomedical applications (Table 1) [13].

Functional silanes were applied as protective agents in the synthesis of many antibiotics (e.g., cephalosporin or penicillin). For example, carbinol groups were reacted with (chloro)trimethylsilane to give a siloxy derivative containing the ≡Si-O-C group, which later underwent a hydrolysis reaction and allowed the recovery of the active drug molecules. Silicones also often serve as useful additives in the pharmaceutical industry [12]. In newer fermentation processes, oxygen was delivered via gas diffusion through relatively thin layers of silicone tubings [55]. Furthermore, it was reported that many pharmaceutical drugs contained small amounts of silicones [61].

The range of medical applications of silicone materials is very wide. By the end of the 1960s, they were employed or evaluated in numerous healthcare applications (Table 2 and Table 3).

The most significant orthopedic applications of silicones are the hand and foot joint implants [62]. The 3D-printed silicon nitride Si_3_N_4_ showed biocompatibility and excellent mechanical, osteogenic, and antibacterial properties. It was considered a very promising material for bone replacement [63,64].

The silicone–urethane copolymer of aromatic polyether and PDMS showed good strength, elasticity, and low adhesion of plasma proteins and fulfilled the bio-engineering requirements [65,66] for use in blood pumps, intra-aortic balloons, total heart replacements, and related devices [13,67]. Carbofunctional hydroxyethyl ether and hydroxypropyl terminated polysiloxanes [68,69] were also used for the preparation of copolymers with aminoalkyl terminal groups [70].

Soluble in water block PDMS–oxyalkylene copolymers formed micelles above the critical concentration and (depending on their structure) at 1 wt.% concentration significantly reduced the surface tension of water [71,72]. Crosslinked polysiloxane–polyoxyalkylene copolymers found medical applications owing to their hydrophilic properties, e.g., in contact lenses [73].

Copolymers of poly(dimethylsiloxane)-α,ω-diols with monofunctional poly(ethylene oxide) (MPEO) of different molecular weights (MWs) (350, 750, and 2000 Da), functionalized with Si(OEt)_3_ groups which were additionally crosslinked with (tetraethoxy)silane Si(OC_2_H_5_)_4_ (TEOS), showed considerably higher hydrophobicity in air. The reduced adsorption of some proteins was observed for these copolymers composed of low-MW PEO chains (350 Da). They were cast in the form of films or processed into different shapes [74]. The permeation of NaCl and glucose through PDMS-poly(*N*-isopropylacrylamide) (PNIPAAm) IPN membranes was studied. They were recommended for practical applications as ophthalmic biomaterials or corneal substitutes [75].

Poly(dimethylsiloxane)-α,ω-diols, diethylene glycol, neopentyl glycol, 1,4-butane-diol, and isophorone diisocyanate were applied for the synthesis of PDMS-based polyurethane prepolymers (PDMS-PUs) with different ratio of soft (SS) and hard (HS) segments. Curing of mixtures of PDMS-PU with hydrophilic monomers: 2-hydroxyethyl methacrylate (HEMA) and N,N-dimethyl acrylamide (DMAA) under ultraviolet (UV) radiation gave PDMS-polyurethane hydrogels, which showed much higher oxygen permeabilities than in the case of silicone-free PU hydrogels and were dependent on water contents. Similarly, the tear strengths and moduli decreased with the increasing water contents of the hydrogels. All PDMS-polyurethane hydrogels exhibited good hydrolytic stability [76].

New synthetic routes and the effect of various starting materials on the morphology, microstructure, and physiochemical properties of polyurethane–siloxane copolymers were described by R. Aeinehvand. The possibility of applications of polyurethanes (PUs) and polyurethane–siloxane biocomposites in hard and soft tissue regeneration, scaffolding, and drug delivery systems and their structure–property relationship was discussed [77]. Other siloxane poly(urethane–urea) copolymers (SiPUU) were prepared from a diisocyanate and a macrodiol which was synthesized in reactions of carbofunctional α,ω-bis(6-hydroxyethoxypropyl) poly(dimethylsiloxane) (PDMS) or poly(hexamethylene oxide) (PHMO) with hexamethylene diisocyanate (HDI) or 4,4′-methylenediphenyl diisocyanate (MDI) or isophorone diisocyanate (IPDI). The SiPUU showed hydrophobic properties (contact angle > 101°) resulting in very low water absorption (0.7% *w*/*w*). Due to several favorable properties, the SiPUU based on MDI and PHMO was suggested to be a perspective biomaterial for the manufacture of synthetic heart valve leaflets [78].

Segmented tough thermoplastic elastomers composed of polyurethane multiblock copolymers containing PDMS and polyether soft segments can be used in medical devices such as leads of pacemakers or defibrillators [79].

The pharmaceutical applications of polysiloxanes, for instance, in skin adhesive patches and controlled drug delivery systems, and the use of silicone additives in the technology of different pharmaceutical products, were described in a review written by Mojsiewicz-Pieńkowska [61].

## 3. Medical Applications of Silicone Elastomers and Rubbers

Silicone rubber (SR) is a common sealing material for a wide range of industrial applications including automotive, medical, electronics, aerospace, and more [80]. SRs are built of seldom crosslinked hybrid polysiloxane chains blended with fillers. They are heat-resistant and inert.

Silicone elastomers (SEs) and SRs show many unique properties (Table 4) and many advantages (Table 5).

Different kinds of SEs and SRs are used for the fabrication of a variety of items including sealants, adhesives, medicine, cooking utensils, thermal insulation, lubricants, electrical insulation hard resins, extrusion profiles, and spreadable fluids. Silicones are highly versatile materials appreciated for their flexibility, biocompatibility, and resistance to temperatures, UV light, radiation, and ozone.

The first silicone elastomer implant in humans was reported by F.H. Lahey who used the so-called “bouncing clay” to repair bile ducts [11]. In 1948, the first human male urethra was replaced with a silicone tube through catheterization [10,13]. In 1968, silicone finger joint implants were developed by A. Swanson. In 1969, silicone was applied for total knee replacement as a shock absorber between the tibial and femoral components [1,8].

Commercial SEs and SRs ensure good quality and reliability. They do not contain solvents, organic plasticizers, phthalates, or latex additives, and are pigmentable and non-blooming in platinum-catalyzed systems. They also show high gas permeability in comparison to most other thermoset elastomers and thermoplastics [89].

The main disadvantages of SR are poor tear strength and a relatively high cost [80].

Liquid silicone rubbers (LSRs) became a basic material for the development and manufacture of medical devices not only due to their outstanding properties, such as biocompatibility, sterilizability, softness and flexibility, and functionality, but also to patient safety and product innovation. The high biocompatibility means that they are well tolerated by the human body and do not cause adverse reactions in contact with living tissues. Somehow, it depends on hydrophobicity, low surface tension, and chemical and thermal stability. This property is crucial for medical devices that come into direct or indirect contact with patients. A useful property of SEs and SRs also specific to medical applications is bio-durability [91,92].

Moreover, SRs show hydrophobicity, low surface tension, and chemical and thermal stability, while hydrophobic (water-repellent) properties of silicones avoid blood coagulation and sticking to wounds. LSRs are nonallergenic and approved for medical applications with skin contact. They are hygienic and inhibit the growth of bacteria and fungi [85].

Silicones are often applied for aesthetic implants in the breast, scrotum, chin, nose, cheek, calf, and buttocks. In some devices, a lightly crosslinked soft silicone gel is used, not containing silica or other fillers, which is swollen with low-MW PDMS oil. The gel is contained inside of elastomer shell in breast, testicular, and chin implants [85].

In the literature, many examples of the biomedical applications of SEs and SRs were described [81,82,83,84,85]. Numerous biomedical applications of silicone elastomers (SEs) and rubbers (SRs) have been known for a long time. Hoses made of SRs, coated inside with heparin which are commonly used for blood transfusion, avoid blood clotting. The first artificial heart and a lot of SR implants have been fabricated so far. Alternatively, siloxane–urethane elastomers can also be applied for this purpose [6]. Crosslinked SEs and SRs, containing reinforcing fillers, usually fumed silica, were used for the fabrication of different medical devices, for example, medical tubing in heart bypass machines, hydrocephalus, pacemaker leads, hydrocephalus, or shunts for the regulation of cerebrospinal brain fluid [13]. Silicone adhesives, gels, and foams are ingredients of various wound dressing compositions that improve the comfort of therapy and decrease nursing costs [93,94,95]. The pure (fillers-free) soft and tacky SEs gels, crosslinked with Pt catalysts, were useful in wound dressings. They show weak adhesion to the skin around a wound and enable the permeability of oxygen and water vapor.

LSRs can be molded into various shapes and sizes in medical devices. Two-part SR systems composed of a liquid base polymer and a crosslinking agent are mixed and processed and they undergo vulcanization, transforming into a solid elastomer with outstanding properties. LSRs are commonly applied in the fabrication of medical implants: catheters, feeding tubes, and pacemaker components, exhibiting biocompatibility and softness. Respiratory masks and tubing made from LSRs are widely used in medical settings thanks to the material’s flexibility and chemical resistance. LSRs are also often employed in the manufacture of surgical instruments: syringe components, surgical grips, and valve seals. Healthcare wearables, such as smart medical patches and monitoring devices, benefit from LSR’s comfortable and skin-friendly properties. LSR gaskets and seals are used to create airtight and watertight seals in medical devices, ensuring their integrity and functionality [91]. LSRs withstand challenges like heat, pressure, and chemical exposure while, at the same time, being comfortable enough for physical contact with patients. The most significant orthopedic applications of silicone are the hand and foot joint implants [92]. The main fields of biomedical applications of SRs are presented in Table 6.

Silicone hydrogels are used for the delivery of pharmaceutical and diagnostic medicines. Owing to their properties, polysiloxanes have encountered a big opportunity as an important material in the area of biomedical engineering with the big influence they have in the area of prosthetic dentistry, tissue engineering, cell growth, and wounded skin treatments, for example, in silicone gel sheets which were clinically proven to rehabilitate hypertrophic scars, and in the design of micropatterning of hydrogels, which were recognized as important bone scaffolds in tissue engineering. They were used for the fabrication of hydrophilic contact lenses as a transfer system for transplanting corneal epithelial sheets to the ocular system, bone joint implants, membranes for gas separation, and heart valve implants and scaffolds. The biocompatibility of this material was improved by the improvement in the surface properties of the polysiloxanes, making them more suitable for cell growth. The ease of crosslinking these materials under normal conditions made them an obvious choice for matrices containing nanoparticles and nanostructures which gave even better properties than the polysiloxanes alone. It was predicted that the polysiloxanes will continue being a reference polymer matrix in biomedical engineering [97].

Silicone implants (SIs) are often used in medical surgeries for the purpose of soft tissue problems. SI is not always completely biocompatible. In order to decrease the number of bacterial and microbial infections on silicone surfaces caused by biofilm growth, many strategies were elaborated which prevent or reduce bacterial adhesion and lead to antibacterial properties. To achieve this goal, different methods were used to oxidize and activate the surface for further modifications, i.e., antibacterial coatings with antibiotics or nanoparticles, covalent grafting of peptides or polymers, and uses of chemicals (acid solutions, sol–gel methods, and chemical vapor deposition), UV, plasma, or ozone. Physical modifications of silicone surfaces, e.g., roughness and topography, were effective as well [98].

The biocompatibility of three facial SEs was evaluated as being good [99]. Very pure, amorphous silica nanoparticles provide an increase in the tensile strength (TS) and tear strength of different kinds of medical-grade SRs. The addition of fillers results in the blood compatibility of silicones. Mainly two types of modified amorphous silica were used as fillers for biocompatible SRs: (1) silica functionalized with reactive Si–H groups (in reaction with MeHSiCl_2_) and (2) silica modified on a surface with covalently bound heparinoid agent exhibiting antithrombogenic properties. Increased albumin binding and biocompatibility were observed for silicones containing hydroxylated and hexadecanoyl groups [100]. The silicone rubber modified with hydrogel coatings composed of poly(ethylene glycol) diacrylate (PEGDA) and poly(vinylpyrrolidone) (PVP), which were thermally cured and showed excellent biocompatibility. The water contact angle (WCA), friction coefficient values, and protein adsorption were significantly decreased. The antifouling and durable lubricious properties were also improved [101].

The polyhydrosilylation reaction of vinyl-functionalized PDMS with poly(methylhydrosiloxane–dimethylsiloxane) copolymer was crosslinked with nontoxic Pt(0) catalyst at mild temperature (within 5 h) and obtained SR gels were used in breast silicone implants [102,103,104]. Chemical risks associated with breast implants were discussed [3].

Attempts at biocompatibility improvement in silicone elastomer were conducted by modification with hyaluronic acid (HA) via polyethylene glycol (PEG) linker, leading to significantly enhanced cell interactions and decreased protein adsorption. Biological interactions of fibrinogen as a model protein with HA-modified silicone surfaces were analyzed. J.G. Alauzun et al. studied the in vitro response of human corneal epithelial cells and fibroblast (3T3) with respect to the starting sample of PDMS elastomer [105].

The biocompatibility and biodegradability of biomedical polymers are important properties for their biomedical applications [106]. The biocompatibility and hydrophobic nature of PDMS, combined with antibacterial surface properties, are crucial for medical applications of silicone implants. PDMS formed smooth surfaces which helped in the osseointegration of implants in the body [107]. Many metals with biomedical purity (e.g., zirconium, titanium, tantalum, niobium, and its alloys) were often added to PDMS implants owing to their biocompatibility [108]. Sol–gel coatings based on PDMS were formed on titanium and stainless steel metallic surfaces [109]. An antibiofouling surface of the hydrophobic PDMS prevented the adhesion of bacteria. It was concluded that for effective applications of implants important are biocompatibility, osseointegration, corrosion resistance, and micro-invasiveness. The polysiloxane hybrid coating material prepared by the hydrolytic condensation of aminofunctional dimethylsiloxane copolymer and tantalum ethoxide which contained Ta_2_O_5_ showed corrosion resistance and biocompatibility properties, which led to great osseointegration [110]. The antibacterial properties of PDMS-based coatings, containing silica, were improved by the addition of 0.5–1.0 wt.% CuO nanoparticles. Moreover, PDMS-SiO_2_ coating filled with 0.5 wt.% CuO significantly promoted MG63 cell proliferation, while the addition of less than 2 wt.% CuO nanoparticles to PDMS-silica coating increased its corrosion resistance [111].

The typical soft SR elastomers prepared by crosslinking linear polymers, PDMS elastomers showed higher storage moduli, significantly less soluble fraction, and significantly lower adhesive properties than crosslinked polysiloxanes with bottle brush structures. In applications as soft materials for biomedical research and engineering, as materials for stretchable electronics, and also in personal care products, the biocompatibility of soft PDMS elastomers is important [112].

Silicone-based bioscaffolds (of brush structures) suitable for cellular therapies were prepared by the crosslinking of α,ω-divinyl(polydimethylsiloxanes) (PDMS) with poly(methylhydrosiloxanes) (PMHS) towards Pt Karstedt’s catalyst. Their physical (i.e., density, elasticity, roughness, wettability, porosity, and pore interconnectivity), chemical, and mechanical properties (tension, hardness, and compression) were studied. Owing to the hydrophobicity of PDMS it is necessary to modify their surfaces (e.g., through oxygen plasma treatment, nanopatterning, etc.) and chemical coating (e.g., polydopamine, fibronectin, etc.) in order to increase their hydrophilicity and cell adhesion to the PDMS surface. The silicone bioscaffold brushes should also be characterized in vivo for biocompatibility (inflammation and vascularization) and in vitro for biodegradation, protein adsorption, cell viability, and morphology. They can be applied in the treatment of type 1 diabetes [9].

Recently, a fully bioresorbable and conductive nerve scaffold integrating N-type silicone (Si) membranes was prepared, which exhibited electrical cues for the repair of nerve defects. This new scaffold was fully biodegradable and enabled accelerated nerve regeneration and motor functional recovery in rodents with sciatic nerve transection injuries. These bioactive materials offer crucial progress for regenerative medicine [113].

Soft polysiloxane microspheres and microcapsules with controlled hydrophobicity and hydrophilicity and different porosity were obtained by the crosslinking of PMHS with (1,3-divinyl)tetramethyldisiloxane in a water emulsion medium. Additionally, a hydrolysis reaction of Si–H groups to ≡Si–OH silanols followed by consecutive dehydrocodensation branching reaction took place. The prepared particles were further functionalized with vinyl, amine, or epoxide functions and modified with hard ceramic microspheres. Further modifications gave the core or shell hybrid inorganic–organic particles of the polysiloxane microcapsules. The prepared original crosslinked polysiloxane microspheres containing Si–H and Si–OH groups were further functionalized via reactions with alkoxy- or chlorosilanes. The incorporation of the (CH_3_)_3_Si– or ≡SiOCH(CH_3_)_2_ groups led to hydrophobic properties, while siloxane moieties were responsible for their hydrophilicity. These particles were used as supports for proteins and Pt and Pd catalysts. Moreover, the microcapsules filled with n-eicosane can be used as phase-change materials or may find numerous biomedical applications [114].

A lipase enzyme was immobilized on room temperature vulcanized (RTV) SRs which were prepared by the crosslinking of PDMS-α,ω-diol with poly(ethylsilicate) and Sn(II) catalyst, and it was modified with aminopropyl vinyl ether (APVE). The SR-lipase biocomposites exhibited over 50-fold increased catalytic activity as compared to the original enzyme [115]. A telechelic vinyl-terminated PDMS was crosslinked with PDMS-PMHS copolymers, treated by plasma, grafted with APVE, and coupled with diazotized heparin or pentafluorobenzaldehyde. These SEs containing covalently bonded heparin can serve as promising novel biomaterials [116]. The SRs prepared by the crosslinking of vinyl-terminated PDMS with PMHS were applied as components of dental products [117]. The SRs with improved physical and chemical properties were used as transdermal, subdermal, and intravaginal devices or in various drug delivery systems [118].

The adhesion of Gram-positive and Gram-negative bacteria to the surface of thin SRs films was decreased by coating with titania and was even strongly reduced by UV irradiation of the TiO_2_ surface layer before contact with the bacteria [119]. The SRs were found to be useful as a filler for balloon catheters and for treating embolisms in kidney arteries. They were applied for preparation of pathological vessels and occlusion of aneurysms. The sterilization of SRs by different methods resulted in their long shelf-life [60].

However, though silicone rubbers are widely applied in medical devices, their low wettability and surface properties are still a challenge in long-term implants. The hydrophilicity of a blend of poly(acrylamide) (PAAm) with the SR was dependent on the PAAm hydrogel loading [120]. A radical grafting of SR-PAAm blend gave soft SR-PAAm composite gels which were useful for medical and biological purposes. Their swelling properties in water solutions were independent of pH value [121]. Surface properties of the SR composite with poly(acrylic acid) (PAA) hydrogel were dependent on hydration time and the equilibrium water content of the PAA hydrogel decreased with increasing crosslinking density [122].

The poly(phenylsiloxane) SR showed very high biostability and high dielectric properties and may be applied as a coating for pacemakers. Silicone–polyurethane thermoplastic copolymers (Cardiothane^TM^, Rimplast^TM^) showed high strength and other good physical properties, blood contacting, and excellent tissue biocompatibility. Their hemocompatibility was improved by grafting hydrophilic vinyl monomers onto silicone surfaces [14,123].

Silicone catheters with long-term antibacterial properties were modified on the surface with antimicrobial poly-L-lysine (PLL) brushes. The silicone catheter modified with PLL showed strong bactericidal properties against pathogens related to catheter associated urinary tract infections. These antibacterial properties were retained after incubating at 60 °C for 65 days or immersing in simulated body fluid for 28 days. Good anti-infection activity and biocompatibility in vivo were also observed during the application of the silicone–PLL catheter [124]. The silicone catheters obtained by surface modification with (3-aminopropyl)triethoxysilane (APTES) and Cu_2_(OH)_3_(NO_3_) nanoparticles showed 99% bactericidal efficacy against *Staphylococcus aureus* and no significant cytotoxicity [125].

The SRs were found to be useful as a filler for balloon catheters and for treating embolisms in kidney arteries. They were applied for preparation of pathological vessels and the occlusion of aneurysms. The sterilization of SRs by different methods resulted in their long shelf-life [60]. Silicone ureteral stents (USs) caused lower body pain intensity (associated with stent removal) in comparison with polyurethane USs [126].

Silicone ureteral stents are the best tolerated and less susceptible to biofilm formation and encrustation on the material from which the stent was made [127].

The widespread applications of SR in medicine such as catheters have several limitations since they show poor tear strength, poor fatigue resistance, and brittle fracture due to poor control of the vulcanization process [128]. It resulted in high failure rates of breast implants and required fabrication under FDA control [129].

However, the properties of silicone rubber catheters were recently improved by graft polymerization of N-vinylpyrrolidone using gamma radiation. These new materials were characterized by ATR-FTIR and Raman spectroscopy, wettability kinetics, TGA, and a drug loading evaluation [130].

Two-component silicone RTV rubber compositions also have great potential as pressure-sensitive adhesives, especially when dedicated to gentle skin adhesives. They exhibited outstanding biocompatibility in in vitro cell culture experiments [131]. Excellent wound healing, angiogenic, and anti-inflammatory properties were observed for the silicone elastomer gel impregnated with 20(S)-protopanaxadiol-loaded nanostructured lipid carriers. It was used for ordered diabetic ulcer recovery [132]. As a drug delivery scaffold, silicone-grafted alginate microcapsules were used for the release of an antidiabetic drug gliclazide [133].

SEs and SRs were recommended for the encapsulation of different types of medical implants and devices [134].

The adhesion of Gram-positive and Gram-negative bacteria to the surface of thin SRs films was decreased by coating with titania and was even strongly reduced by UV irradiation of the TiO_2_ surface layer before contact with the bacteria [119]. In order to enhance the surface properties of the commercial SR and keep its transparency in the visible spectrum, they were also modified by incorporating amorphous TiO_2_ into the silicone matrix through the sol–gel method using titanium (IV) ethoxide. A transparent PDMS elastomer with increased surface hardness and biocompatibility was obtained. It showed good antibacterial and antifouling properties, lower coefficient of friction, complete removal of tackiness, and a glassier surface. The transparency was maintained and the surface hardness was significantly increased, which was promising for various biomedical applications [135].

The application of silicone gel combined with antibiotic dressings reduced the inflammatory response of rats’ skin and increased the healing index [136]. A new sterilization method, ozonation (instead of gamma irradiation and steam heat) of a silicone hydrogel based on tris(trimethylsiloxy)silyl] propyl methacrylate, gave the biomaterial with profitable intrinsic properties. This material was designated for the preparation of soft contact lenses and their ophthalmologic applications [137]. It can be summarized that owing to outstanding properties: biocompatibility, chemical resistance, flexibility, etc., LSRs have found numerous medical applications: from implants and surgical instruments to healthcare wearables and respiratory devices [138]. In order to fulfill all the medical restrictions, manufacturers of LSR medical devices must fulfill ISO 13485 standards [139] and FDA regulations throughout the design, production, and distribution process of these biomaterials [91]. 

### Examples of 3D Printing of Silicone Elastomers for Biomedical Applications

Poly(siloxane–urethane) elastomers (PDMS-PUs) with good thermally self-healing properties were prepared with telechelic PDMS terminated with isocyanate groups (as soft segments) or its mixture with polycaprolactone diol (PCL). The incorporation of the PCL segments into the PDMS-PU chain resulted in the enhancement of their mechanical properties. The fairly good biocompatibility of the PDMS-PU elastomers was confirmed by animal wound healing experiments and cytotoxicity tests. They can find potential biomedical applications, e.g., artificial skin having self-healing properties [140]. The application of the 3D printing of PDMS-PU elastomers was very useful for the preparation of medical devices [141].

A new silicone elastomer chain that can self-assemble into a 3D periodic structure was prepared, in which PDMS chains were terminated with self-assembling triptycene molecules. These novel soft materials should find optical, mechanical, heat/charge transportation, nanotechnological, and biomedical applications [92]. Such ordered structures self-assemble into the desired shape [142]. The 3D-printed silicone elastomers were characterized by various methods by Miron et al. [143].

By the thiol-ene photopolymerization of vinyl-terminated PDMS and thiol-functionalized polysiloxane silicone wound dressing was prepared, which was UV-cured and 3D printed. The gelation time of this biocompatible silicone elastomer was dependent on the thiol-ene molar ratio and time of UV irradiation intensity. It showed antibacterial properties against Gram-positive (*S. aureus*) and Gram-negative (*E. coli*) bacteria, cytotoxicity, and biocompatibility, and was conducive to the recovery of wounds. The thiol-ene UV-curable silicone elastomer can be a promising material in biomedical applications such as wound dressings or customized soft tissue scaffolds via 3D printing into various shapes with smooth surfaces and good precision [144].

Different kinds of silicone-based biomaterials (adhesives, fillers, elastomers, gels, and many others) were prepared from silicone polymers, quite often with the addition of nanosilica and silane coupling agents. They have significant therapeutic potential and future perspectives, especially due to safety, biocompatibility, good quality of the silicone implants, and the growing progress in silicone-based 3D bioprinting and tissue engineering [145].

Droplet-based, extrusion-based, or laser-based material deposition methods are used in 3D printing. For complex silicone-based elastomers, processing problems mainly concern their low elastic modules. A hydrophilic gel of crosslinked polyacrylic acid was used as a processing aid [146]. After curing, the gel was removed by washing with a phosphate saline solution. Helical and cylindrical tubes and PDMS elastomer cuffs for the wearable pulse oximeter were prepared [147]. Patients fitted with 3D-printed prostheses had better hand performance than those without aid [148]. The direct silicone printing of facial silicone prostheses was achieved with four nozzles [149]. The auricular prosthesis made of silicone with various grades of flexibility and hardness enabled patients’ effective rehabilitation and fitted well with their anatomy [1].

**Figure 2:**
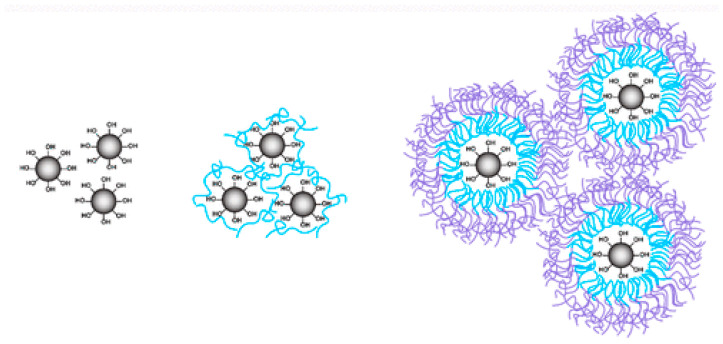
Silicone printable inks modified with silica fillers (**left**) and thixotropic additives: PEG (**middle**) and amphiphilic agents (**right**). Reprinted (adapted) with permission from [5]. Copyright (2023) American Chemical Society. Licensed under CC-BY 4.0.

The method of 3D printing using silicone inks with high viscosity caused problems with mixing. The use of low-viscosity silicone inks and a single nozzle followed by rapid vulcanization allowed the fabrication of soft elastomeric materials [150]. Rapid crosslinking under UV irradiation (within seconds) eliminated the use of support material and led to excellent adhesion between printed layers. In finger joint implants, 3D printing allows the silicone spacer’s complex design to distribute a better load fitted to each patient’s unique condition. The silicone-based bioprinting is still in the early stage and requires further development [1]. During 3D printing, the silicone ink must undergo fluidization at high shear and stiffening at low shear or rest. An improved printability of silicones was achieved by the application of rheological modifiers, e.g., nanosilica and thixotropic additives (Figure 2) [5].

## 4. Other Biomedical and Cosmetic Applications of Silicones and Modified Silica

Many silicone ingredients exist in cosmetics as well as other areas such as healthcare, aerospace, electronics, transportation, construction, and energy. Approximately 50% of the new personal care products contain at least one type of silicone. Silicones from cosmetic products might reach municipal wastewater treatment plants, where silicones form sludge and have no negative effects since it is so insoluble in water. Subsequently, sludge is burned, buried in a landfill, or applied as fertilizer to fields of crops. Furthermore, silicones permeate the soil ecosystem and are degraded by soil hydrolysis. The volatile silicone components in the upper atmosphere, where hydroxyl radicals break the Si–C bonds, release silica, water, and carbonyl compounds, which are part of our ecosystem. The rate of silicone degradation is remarkably rapid. The hydrolytic degradation in dry soil takes 4–7 days, while volatile silicones degrade within 10–30 days. Silicone polymers or their decomposition products do not accumulate in the environment and are not harmful to land or aquatic life or plants [151].

**Figure 3:**
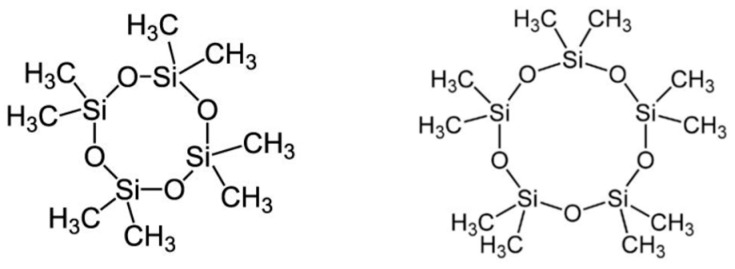
Chemical structures of D_4_ and D_5_.

Octamethylcyclotetrasiloxane (Me_2_SiO)_4_ (where: Me = CH_3_) (D_4_) and decamethylcyclopentasiloxane (Me_2_SiO)_5_ (D_5_), called *cyclomethicones*, are most often used dimethylsiloxane oligomers in cosmetic and personal hygiene products (Figure 3).

Many hair and skin formulations are based on functional silicones, especially composed of amine or polyether moieties as ingredients. Poly(dimethylsiloxane-α,ω-diols) of the general structure HO(Me_2_Si)_n_OH (called *dimethiconols*) form water-resistant films which are ingredients of skincare, sunscreen, or decorative cosmetics [152]. Polymethylsiloxanes containing aminopropyl side groups (called *amodimethicone*) are important components of cosmetic goods, and particularly, hair conditioners [153]. These groups protect hair color against degradation owing to frequent head washing or under exposure to UV radiation, and enable a reduction in drying time without negative effects on hair volume and structure. Moreover, the antistatic effect is observed for products containing amodimethicone [154].

Blends of PDMS with UV-B additives such as p-aminobenzoic acid derivatives or (p-methoxy)cinnamates are used as sun tan lotions. Many applications in the fabrication of personal care and cosmetics products, and also in versatile aesthetics formulations find carbofunctional silicones [155,156,157,158,159]. However, in recent decades, an increasing interest in the application of natural products in the cosmetic field has been observed [160].

**Figure 4:**
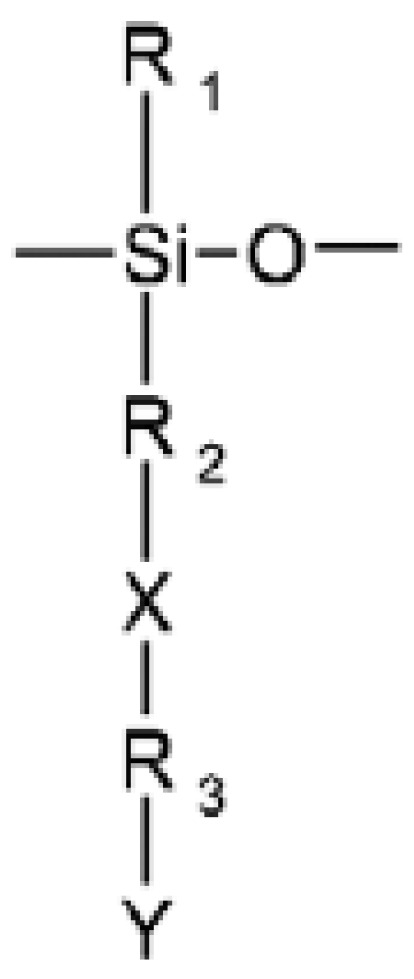
The general chemical structure of oligosiloxanes and polysiloxanes having good anti- microbial properties; where R_1_, R_2_, and R_3_—alkyl or alkylene groups containing 1–10 or 1–18 C atoms, X and Y—alkyl or aryl quaternary amido or imido groups, containing 1–20 C atoms [161].

Good antimicrobial properties showed oligosiloxanes and polysiloxanes containing alkyl or aryl quaternary amido or imido groups. Their chemical structure is presented in Figure 4.

Silicone gels and silicone gel sheets have been used in scar therapy for many years. In dermatological products, silicone polymers of different types and structures are used [162]. For example, a graft polysiloxane copolymer with t-butylaminoethyl methacrylate (containing a quaternary ammonium group) having covalently attached fluorescein was easily miscible with SEs and exhibited good antibacterial properties with respect to Gram-negative bacteria *Escherichia coli* [163]. A polysiloxane containing 5,5-dimethylhydantoin-based N-halamine pendant groups was prepared by the alcoholysis of N-halamine precursor 3-(3-hydroxypropyl)-5,5-dimethylhydantoin and the subsequent chlorination of hydantoin ring with t-butyl hypochlorite. The polyethylene (PE) fibers impregnated with N-halamine-grafted polysiloxane gave a thin coating layer in supercritical CO_2_ and showed antibacterial properties against *Escherichia coli* and *Staphylococcus aureus*. Such coating layer on PE fibers was stable during storage, washing cycles, and UV irradiation [164].

Silicone-modified block copolymers showed good compatibility with polypropylene (PP). Compositions of styrene–butadiene–styrene block copolymer (SEBS) blended with PP and silicone show exceptionally profitable properties for biomedical applications. They have very good elastomeric and hydrophobic properties and do not contain phthalate plasticizers. Silicone-based biomaterials and hydrogels have easily wettable surfaces depending on the content of the hydrogel phase.

An ion implantation technique was useful for improvement in silicone antithrombogenicity. Plasma treatment caused an increase in silicone hydrophobicity. Amine-functionalized silica nanoparticles of three different average diameters (50, 100, and 200 nm) were prepared by a reverse microemulsion method [165]. Silica nanoparticles (NPs) and nanorods were functionalized with amino(alkoxy)silanes, followed by modification with N-diazeniumdiolate nitric oxide (NO) donors. They were useful for the fabrication of NO-releasing materials, which showed antibacterial properties. The hybrid nanoparticles, prepared with the use of TEOS and N-(6-aminohexyl)aminopropyl(trimethoxy)silane, had a high monodisperse size. The antibacterial efficiency of the NPs releasing NO was higher for their smaller particles [166,167].

Mesoporous silica nanoparticles, modified with chitosan, formed hydrogen bonds between the –NH_2_ group of chitosan and –OH groups of NPs and were used as bio- degradable pH-responsive nanocarriers of drugs [131]. The mesoporous silica (MCM-41) was used for the adsorption and release of Naproxen. The MCM-41 silica had a regular structure of pores, a high pore volume (~1–2 cm^3^/g), and a high specific surface area (~1000 m^2^/g) [168].

## 5. Biomedical Applications of Polymeric Nanocomposites Containing POSS Nanofillers

Polysiloxanes and other silicone polymers are universal materials used in high-tech technologies and modern materials [11]. Silsesquioxanes (POSS compounds) contain the smallest possible silica skeleton and their molecules with model nanosilica structures and are applied as “nano-building” units for the preparation of inorganic–organic hybrid materials. They are nontoxic and show good stability and biocompatibility. POSS nanoparticles were used as fillers in hydrogels having the processability of organic materials and the rigidity of inorganic materials. The excellent POSS-based hybrid hydrogels were used for bone regeneration, in cell culture, and in drug delivery systems [169].

Polymeric composites containing POSS have found biomedical applications due to their biostability and nontoxicity [170]. For example, ammonium-functionalized quaternary silsesquioxanes (Q-POSSs) were obtained by the hydrosilylation of octakis(hydrodimethylsiloxy)octasilsesquioxane [(HMe_2_SiO)SiO_1.5_)_8_] with allyldimethylamine. Compositions containing Q-POSS exhibited antimicrobial activity against the Gram-positive bacterium *Staphylococcus aureus* and the Gram-negative bacterium *Escherichia coli*. The antimicrobial activity of elastomeric polysiloxane coatings was dependent on a Q-POSS structure. The Q-POSS with a relatively low quaternization and longer alkyl chains showed the highest antimicrobial activity in solution. Unexpectedly, the coatings based on Q-POSS having the highest quaternization level did not exhibit antimicrobial properties, probably due to the agglomeration of Q-POSS molecules, which reduced diffusion and inhibited the inter- action of the quaternary ammonium POSS halides with microbial cells [171]. The crosslinked copolymers of PEG with POSS formed a hybrid cement having a similar strength to commercial bone cement [172].

Eight-arm silsesquioxane macromonomer, which contains vinyl-terminated segments of PEG 400 in a corner, is water-soluble and forms spherical aggregates in aqueous solution above the critical micelle concentration (CMC), as it was confirmed by transmission electron microscopy (TEM) pictures. It was copolymerized with a triblock copolymer of lactide–PEG–lactide having vinyl end groups in a mixture of acetone and water (1:4 *v*/*v*) to yield crosslinked hydrogels. The prepared porous copolymer networks showed swelling properties and at pH 7.4 underwent a slow hydrolysis with the evolution of lactic acid and PEG. These hydrogels were noncytotoxic toward fibroblast cultures and can be applied as scaffolds for bone repair [173].

By grafting hydrophobic POSS molecules onto hydrophilic star PEG-maleimide resin, other hydrolytically degradable POSS–PEG hybrid hydrogels were prepared. The obtained precursor was next crosslinked with PEG-diester-dithiol in an aqueous medium. The POSS–PEG hybrid hydrogels showed a porous structure and high hydrophilic properties. Their swelling ratio, degradation rate, and mechanical properties were tuned by the grafted hydrophobic POSS [174].

Inorganic–organic oligomeric POSS–PEG hybrid hydrogels were obtained by grafting POSS molecules onto 4-arm-PEG-maleimide in a water medium. They were next crosslinked with matrix metalloproteinase (MMP) peptide. The POSS–PEG hybrid hydrogels had a porous structure and highly hydrophilic properties and were biodegradable with human MMP-2 solution. The degradation rate was dependent on the POSS content. The POSS–PEG hybrid hydrogels had good biocompatibility and were recommended for tissue engineering scaffolds [175].

The hydrolysis of acetoxyphenyl-functionalized POSS molecules gave octa-, deca-, and dodecahydroxy-substituted cubes, which were next treated with adipic acid chloride and gave highly crosslinked POSS-polyester copolymers. These materials, having specific surface areas of 5–25 m^2^/g, may find various applications, e.g., in drug delivery systems [176].

The hydrosilylation of telechelic vinyl functionalized PLLA macromolecules (mPLLA) with octakis(hydrodimethylsiloxy)silsesquioxane gave a series of hybrid star PLLA (sPLLA) structures. The prepared sPLLA had five to seven arms of sPLLA. Their T_g_s and T_m_s values were higher than those of the mPLLA. High-resolution TEM pictures indicated that POSS units formed 5–20 nm aggregates in the crystalline PLLA matrix. Microspheres of sPLLA (with a diameter of 1 to 2 μm) were used in drug delivery systems [177].

Polylactide (PLLA) scaffolds containing 0–5 wt.% POSS and functionalized with PEG showed cytocompatibility (with human stem cells) and did not show any cytotoxicity. The PEG-POSS nanoparticles (NPs) were well dispersed in PLLA and caused an increase in the specific surface area and a decrease in mean fiber diameter. The decrease of the hydrolytic degradation rate of the PLLA/PEG-POSS nanocomposites (NCs) was also observed. The new PLLA/PEG-POSS NCs can serve as a potential biomaterial for cartilage regeneration [178].

New star-shaped poly-L-lactide-PEG-POSS (SPPS) hybrid membranes were prepared by a photocrosslinking of methacrylate POSS with (methacryl)urethane functionalized star poly-L-lactide and poly(ethylene glycol) diacrylate. Inositol was used as a core. The SPPS had tunable mechanical properties (5.8–130 MPa in tensile modulus, 30–144% in elongation, over 90% recovery), osteoblast biocompatibility, good biomineralization activity, and biodegradation. Owing to their good properties, these multiarmed bio- degradable membrane hybrids may find biomedical applications, e.g., in bone tissue regeneration [179].

By grafting amphiphilic poly(L-aspartate)-*b*-poly(ethylene glycol) block copolymers on carboxy-functionalized POSS, other star-shaped copolymers were prepared. 1-(3-Aminopropyl) imidazole formed pH-sensitive micelles with poly(L-aspartate) segments, having diameters in the range of 50–60 nm. They were applied to trap the anticancer drug doxorubicin (DOX) in the micelles. The release of the drug micelles containing imidazole groups was affected by pH. At pH 5.0, over 90% of the DOX was released within 48 h. It was reported that these micelles exhibited good anticancer properties [180].

Single-walled carbon nanotubes (SWNTs) were modified with amine-functionalized POSS molecules dispersed in water or in organic solutions. They showed very low toxicity and were recommended for biomedical applications [181]. Unlike carbon nanotubes [182], due to cytocompatible features and a viscoelastic effect, NCs containing POSS molecules can be applied for tissue engineering and vascular prostheses [183,184]. However, cell adhesion, viability, and proliferation between POSS NCs and standard cell culture plates were similar [185,186]. The unique properties of the polymer-POSS composites are important for their future applications as biological and medical devices, particularly in cardiovascular bypass grafts [182].

The nontoxicity to biostability of POSS and POSS-based polymers increased their biomedical applications [187]. Moreover, the NCs composed of POSS molecules showed profitable mechanical properties, good hydrolytic and oxidative resistance, biocompatibility and biostability, resistance to calcification and fatigue, endothelialization properties, reduced in vitro inflammatory response, and antithrombogenic potential. The NCs containing POSS nanofillers should find many biomedical applications, for example, as biosensors, cardiovascular and breast implants, dental materials, drug delivery systems, and in tissue engineering. The POSS polymers containing Ag nanoparticles showed improved wound healing [188] and excellent anti-inflammatory activity [189].

POSS-modified materials were also used as coatings for greenhouse covers and sun protection agents which absorb UV light and reduce skin cancer [190,191,192,193]. Poly(vinyl silsesquioxanes) showed greater photochemical and thermal stability than organic compounds used in sunscreen formulations. Films of vinyl silsesquioxanes oligomer (VS) obtained by the sol–gel polycondensation of vinyl(trimethoxy)silane (VMS) were thermally cured with benzoyl peroxide as an initiator. Similarly, coatings of vs. hybrids with silica (VSTE) were obtained from VMS and 5–25 wt.% (tetraethoxy)silane and hybrids films of vs. with titania (VSTT) were obtained from VMS and 5–25 wt.% titanium tetrabutoxide. The following experimental transparencies were measured for the modified films: 9–14% between 280 and 320 nm, 67–73% between 320 and 350 nm, and 86–89% between 350 and 400 nm. VS, VSTE, and VSTT had good absorption in the UV-C and UV-B ranges, but did not absorb UV-A. The UV-B absorption was not improved by the addition of SiO_2_ or TiO_2_, but the transparency of thin films to UV was increased [194].

Trisilanol isooctyl-POSS metalized with titanium nanoparticles was used for the modification of a polycarbonate (PC)–urethane copolymer, which showed better mechanical and thermal properties in comparison to pristine POSS and also affected the surface properties. Owing to its hydrophobicity and biocompatibility cell viability was improved, which should increase their potential biological and medical applications [195].

PEG, POSS, TiO_2_, titanium, titanium nitride, heparin, F-heparin, vinyl pyrrolidone, 2-methacryloyloxyethyl phosphorylcholine, and inhibitors of cytokines were used for the modification of the surface properties of intraocular lenses. Better biocompatibility was observed for the obtained lenses with a hydrophilic anterior and hydrophobic posterior surface [196].

Octa(3-chloroammoniumpropyl)octasilsesquioxane (T_8_-POSS) was found to be a promising nanocarrier for anticancer drug doxorubicin (DOX) delivery. The cytotoxicity of DOX and T_8_-POSS-DOX system toward breast cells (MCF-7), microvascular endothelial cells (HMEC-1), and human cervical cancer endothelial cells (HeLa) was determined. The T_8_-POSS-DOX complex at the molar ratio of 1:8 was more effective than free DOX [197]. W.A. Stańczyk et al. studied the impact of POSS on mammalian cells. Studies in vitro of the cytotoxicity of octaammonium chloride salt of octaaminopropyl POSS toward two cell lines—embryonic mouse hippocampal cells (mHippoE-18) and mouse neuroblastoma (N2a)—revealed no detrimental effect on both cells. The noncytotoxic property of POSS opened the possibility of this kind of biocidal POSS application as a promising drug nanocarrier [198]. Attempts to use NCs, based on functionalized POSS molecules, dendrimers, and hybrid organo-inorganic copolymers as potential supports in modern theranostics and for the encapsulation of bioactive compounds were described. Conjugates formed by functional silsesquioxane structures: octa(3-chloroammoniumpropyl)silsesquioxane or octa(carboxydecylthioethyl)silsesqui oxane with anticancer anthracyclines were used for this purpose [199,200].

The addition of cationic octaammonium (OA-POSS) nanoparticles into chemically crosslinked cationic poly(dimethylaminoethyl methacrylate) hydrogels using radical freezing polymerization gave new kinds of hydrogels (OP-PD gels). They had significantly improved mechanical properties: higher tensile and compressive strength. Due to universal swelling properties, OP-PD gels can be used in controlled drug release [201].

POSS-poly(carbonate-*co*-urea)urethane was obtained from polycarbonate polyol, *trans*-cyclohexanediol heptaisobutylsilsesquioxane, methylene diisocyanate, ethylenediamine, and diethylamine in N,N′-dimethylacetamide solution. Owing to its antithrombogenic properties, they can find applications as the microvascular component of artificial capillary beds and in cardiovascular bypass grafts [202].

Recently, growing interest was observed in POSS-poly(carbonate-urea) urethane (POSS-PCU) as a new versatile nanocomposite biomaterial, e.g., as a bioscaffold for tissue engineering. The properties of POSS-PCU were improved by the addition of porogens (Na_2_CO_3_, NaHCO_3_, NaCl, and saccharose) onto the material surface, which resulted in an increase in surface porosity. Porogens affected the mechanical strength, wettability, surface chemistry, and cytocompatibility of the composite material. For the surface modifications of POSS-PCU scaffolds (with a focus on in vitro cytocompatibility), primary human bronchial epithelial cells were used. As a result of the grafting of the POSS-PCU scaffold in vivo, it was noticed that bigger pores favored cellular integration and vascular growth [203].

Nanohybrid polyurethanes modified with POSS nanofillers can be used as medical implants of improved biological resistance, and also as attractive materials in material engineering, microelectronics, optics, etc. [204,205].

Newer dental resin composites, based on methacryl functionalized resins, contained different amounts of nanosilica and (octamethacryl)octasilsesquioxane (POSS), and were cured with the use of UV irradiation. Due to the presence of POSS, the volume shrinkage of these composites was decreased, while their mechanical properties were initially increased [206].

## 6. Applications of Silane and Siloxane-Modified Thermoplastics in Medicine

Silica and silane-modified thermoplastics also find many applications in medicine. Different reactive silanes containing carbofunctional groups (CFSs) were useful in the fabrication of contact lenses. The prepared special membranes pass oxygen which is measured as the equivalent oxygen percentage (EPO). The minimum EPO value equals 5–7%. In soft PDMS-based lenses with a thickness of 0.2 mm, the EPO value reached ~20%. The lenses made of pure PDMS elastomer are not used owing to the hydrophobic properties of PDMS, which could cause damage to the cornea. The silanes having carbofunctional groups or/and silicone–methacrylic copolymers: (methacryloxypropyl)(trimethylsiloxy)silane (TRIS) or (methacryloxypropyl)pentamethyldisiloxane are most often used for their preparation. The EPO values for the contact lenses made from such materials achieve only ~10% [207].

Hydrogels have been used for a long time in pharmaceutical and biomedical applications, for example, a biocompatible superadsorbent hydrogel of poly(2-hydroxyethyl methacrylate) (PHEMA) was used in the preparation of ophthalmic contact or intraocular lenses, soft-tissue exchange, vascular prostheses, and drug delivery. Bioactive PHEMA-silica hybrids were prepared by the addition of silica NPs to a solution of 2-hydroxyethyl methacrylate (HEMA) monomer with PHEMA or the solution of (tetraethoxy)silane Si(OEt)_4_ (where: Et = C_2_H_5_) (TEOS) and HEMA. Copolymers of HEMA and 3-aminopropyl(triethoxy)silane (APTES) were prepared by the hydrolytic condensation of TEOS, while HEMA units were radically polymerized in situ. The obtained hybrid biomaterials were applied as bio- scaffolds for bone engineering [208].

PLA with biocompatible and biodegradable properties has also found various biomedical applications. Triblock copolymers of poly(lactide-*b*-methylvinylsiloxane-*b*-lactide) were bonded with magnetic NPs. The obtained magnetic nanocomposites were applied in drug delivery, magnetic hypertherma tumors therapy, and magnetic cell separations [209].

It is well known that polysiloxanes, modified with quaternary ammonium salt (QAS) groups, showed good antibacterial and antifungal properties. Antimicrobial hybrid siloxane–epoxy coatings containing QAS moieties showed self-decontaminating properties, which reduced up to 99.9% of pathogenic bacteria on the surface [210].

Polyurethane elastomers find biomedical applications owing to their perfect mechanical properties, good hydrolytic and oxidative resistance, good processability, and biocompatibility. Most often, PDMS copolymers containing polyurethanes (PUs) or polyureas (PUU) blocks are based on polycarbonate or polyether soft segments. One of the commercial functionalized copolymers, poly(ether–urethane), contained ~10 wt.% PDMS soft segments. These copolymers can be used for the fabrication of implants, intra-aortic balloons, vascular grafts, artificial heart grafts, etc. [211].

A composite silicone rubber–polyacrylamide (PAAm) hydrogel exhibited a pH-independent swelling degree, nonionogenic character, and had high specific surface area. The composites of hydrogels with SRs were often used in drug release systems [118,212].

Polysiloxane copolymers having side glucosylthioureylene groups may be applied as biomaterials and as model amphiphilic polymers, e.g., polysiloxane–saccharide copolymers: PDMS-grafted saccharose or PDMS-grafted amylase. These simple models of biopolymers grafted with oligosaccharides were used as artificial glycoconjugates in medicine and biochemistry [213].

Different siloxane-based compounds and polymers may be applied for the preparation of medical implants. For instance, (octaphenyl)cyclotetrasiloxane or poly(mercaptopropylmethylsiloxane) were used for coating the gold electrodes of acoustic wave sensors. The irreversible adsorption of various proteins (human serum albumin, fibrinogen, hemoglobin, immunoglobulin G, cytochrome C, and others) to both siloxane surfaces and a gold electrode was observed [214].

An inorganic–organic hybrid epoxy–silica material containing 50 wt.% SiO_2_ was obtained in two steps: (1) by the reaction of bisphenol A diglycidyl ether with APTES, followed by (2) the hydrolytic polycondensation with Si(OEt)_4_ in THF. The powdered solid hybrid epoxy-SiO_2_ nanocomposite (NC) was used as an ingredient of bone cement. The liquid components were composed of bis-phenol-A glycidyl methacrylate, triethyleneglycol dimethacrylate, and 20, 25, or 55 vol.% of methyl methacrylate (MMA), respectively. The mechanical properties of the epoxy–SiO_2_ NC were better than those of commercial bone cement, while the tensile and bending strengths were similar for both cements. The new epoxy–SiO_2_ composite showed a very low cytotoxicity in comparison with a similar commercial product [215]. So-called PDMS-TEOS *ormosils* with various contents of Ti and Ca exhibited in vitro bioactivity, which depended on the presence of highly polar surface properties and specific roughness [216].

A pore- and crack-free transparent monolithic organic–inorganic hybrid material composed of a Si–O–Ti–O–Ca network modified with methyl groups was prepared by the hydrolytic polycondensation of HO(Me_2_SiO)_n_H with TEOS and (tetraisopropyl)titanate in the presence of Ca(NO_3_)_2_. This hybrid material showed good mechanical properties similar to those of the human cancellous bone, and high bioactivity. It can be used as a new bone-repairing biomaterial [217].

CaO–SiO_2_–PDMS inorganic–organic hybrid coatings, obtained by a sol−gel dip-coating method, over Ti_6_Al_4_V substrates also showed bioactive properties. These hybrid coatings are important for potential orthopedic and dentistry industries applications [218].

An aqueous solution containing 30–40 wt.% of aminoalcohol hydrochloride or choline chloride and alkali metal chloride and 8.0–12.5 wt.% oligomers of orthosilicic acid with the general formula [(≡SiO)_n_]Si(OH)_4–n_(OH)_3n_ (n = 2 or 3) showed bioactive properties [219].

Homogeneous PVA-PDMS microsphere composite hydrogels were characterized by a relatively high swelling ratio and equilibrium water content, enhanced water retention, and compressive modulus. It can find application as wound dressings [220]. A silicone network of the new hydrogel material was obtained by the sequential interpenetrating network method from Si–H and Si–vinyl functional siloxane oligomers. A hydrophilic network of this hydrogel was prepared by the radical copolymerization of N-vinyl pyrrolidone and N,N′-dimethylacrylamide with a crosslinking agent (ethylene glycol dimethacrylate). The hydrated hydrogel was transparent and hydrophilic; showed high mechanical properties, good wettability, and oxygen and water permeability; and can be useful for medical applications [221].

By the copolymerization of poly(N-isopropylacrylamide) (PNIPAAm) with 3-methacryloxypropyl(triethoxy)silane, thermoresponsive shape memory polymers, which are soluble in water, were prepared. They should find biological and medical applications [222]. A self-assembly silane monolayer was obtained by the graft polymerization of N-isopropylacrylamide (NIPAAm) with APTES on the glass surface. The glasses coated with NIPAAm-based films can be used as environmentally switchable materials [223]. The amphiphilic block copolymers of a PDMAAm-*b*-PDMS-*b*-PDMAAm structure containing poly(N,N-dimethylacrylamide) (PDMAAm) and PDMS blocks should also find numerous medical applications [224]. The triblock copolymer of PDMS with PMMA [poly(methyl methacrylate)] and PHFBMA (2,2,3,3,4,4,4-heptafluorobutyl methacrylate), with a very low surface energy, can find applications as antifouling coatings [225].

The copolymers which showed hydrophilicity, high oxygen permeability, and transparency, necessary for contact lenses (CLs) applications, were synthesized by the radical polymerization of amphiphilic fumarate monomers (derivatives of styrene and N-vinylpyrrolidone, NVP) containing hydrophobic and hydrophilic groups: 2-(2-methoxyethoxy)ethyl, 2-(2-(2-methoxyethoxy)ethoxy)ethyl, and 3-[tris(trimethylsiloxy)silyl]propyl. Different membranes, obtained by the copolymerization of these fumarate monomers with NVP or NVP and HEMA, showed much better transparency in comparison with the membranes that contained 3-[tris(trimethylsilyloxy)silyl]propyl methacrylate units. Above polyfumarates with silyl substituents can be applied as oxygen-permeable membranes for contact lenses (CLs), crystal liquids, high-performance biodegradable materials, low dielectric materials, etc. The CL materials should exhibit not only oxygen-permeability for the breathing of corneal cells but also hydrophilic properties for not repelling a tear and to protect the deposition of lipids [226].

Poly(ϵ-caprolactone) (PCL)-based shape memory polymers (SMPs) show biocompatibility, biodegradability, and elasticity of PCL, and a well-defined T_m_ in the range of 45–60 °C with increasing M_n_. The properties of PCL-based SMP networks were modified through the incorporation of organic hard segments (HSs) or soft segments (SSs). PDMS with extremely low T_g_ (−123 °C) was found to be a very effective SS component. Inorganic–organic shape memory polymers (SMPs) containing PDMS soft segments (having very low T_g_, −123 °C) with aminopropyl end groups and organic poly(ϵ-caprolactone) segments showed splendid shape recovery and fixity. Their mechanical, thermal, and surface properties were tailored by changing the length of the PDMS blocks [227].

PLLA blends with different contents of biocompatible methyl silicone resins (Me-MQ, where M is the siloxane unit R_3_SiO_1/2_ and Q is the tetrafunctional siloxane unit SiO_4/2_) were obtained after solvent removal. The elongation at break of the PLLA blends increased to 67.9% and 217.7% at the loadings of 1 and 2 wt.% Me-MQ, respectively, without a decrease in their tensile strength [228].

Examples of the biomedical applications of silicones and silicone–urethanes (e.g., as artificial skin, breast implants, interactive balloon pumps, and contact lenses) were reported a long time ago by B. Arkles) [207]. They were also useful for fabrication of heart valves [78]. PDMS-diols or hydroxyalkenyl or aminopropyl or ethylpiperazine- terminated PDMS or poly(alkenyl oxide)s were often used as soft segment (SS) components in PDMS copolymers with polyurethanes and polyurethaneureas. The solubility parameter of poly(propylene oxide) (PPO) (23.5 J^1/2^/cm^3/2^) is in between that of PDMS (15.6 J^1/2^/cm^3/2^) and urea (45.6 J^1/2^/cm^3/2^), which affected the nature of the interphase between the soft matrix and the hard urea microdomains and the intersegmental mixing between the PPO and urea segments [229].

By the ATRP method, random and block polyethylene glycol (PEG)-*co*-PDMS brushes were prepared from (monomethacryloxy)propylterminated PDMS (6–9 cSt) and oligo(ethylene glycol) methyl ether methacrylate (M_n_ = 475 g/mol). PEG-*co*-PDMS brushes were nonadhesive in water, but they showed biocompatibility and can be applied in cosmetic formulations [230]. By the ATRP of perfluorohexylethyl polyacrylate (FA) monomer, well-defined amphiphilic diblock copolymers EG_x_-FA_z_ and pentablock copolymers FA_z_-EG_x_-(PDMS)_y_-EG_x_-FA_z_ containing polysiloxane (PDMS), PEG, and FA blocks were prepared. These block copolymers were resistant to protein adhesion, but their blends with PDMS led to increased adsorption of proteins, while the PDMS matrix films were able to effectively remove sporelings of the green fouling alga *Ulva linza*. They were also used for the fabrication of coatings with antibiofouling properties [231].

Random and block copolymers of methyl methacrylate (MMA) and 3-(heptamethyl cyclotetrasiloxanyl)propyl methacrylate (HCPM) had two distinct T_g_s. The gas permeability of oxygen and nitrogen for MTTS-MMA random copolymers prevailed over those of tris(trimethylsiloxy)silylmethacrylate (MTTS) copolymers. The diffusion and solubility coefficients depended on the content of HCPM mers and the crosslinking degree [232].

Polyurethane (PU) hydrogels were prepared in two steps from polysiloxane-based PU prepolymers and hydrophilic monomers. The PU prepolymers containing hard and soft segments were synthesized with the use of the following monomers: isophorone diisocyanate, neopentyl glycol, diethylene glycol, 1,4-butanediol, telechelic PDMS-diol, and 2-hydroxyethyl methacrylates (PHEMA). As the hydrophilic monomers, the following were used: 2-hydroxyethyl methacrylate (HEMA) and *N*,*N*-dimethyl acrylamide. All the compositions were cured with UV light and formed hydrogels after hydration. Their tear strengths and moduli, and oxygen permeabilities decreased with the increasing water contents. The oxygen permeabilities of hydrogels were much higher than those of non-silicone hydrogels. These PU hydrogels had good hydrolytic stability and did not absorb lysozyme [76]. Thermoplastic PU–siloxane copolymers (PU-Ss) were synthesized from 4,4′-methylenediphenyl diisocyanate (MDI) and 1,4-butanediol and 40–90 wt.% of poly(propylene glycol)-*b*-poly(dimethylsiloxane)-*b*-poly(propylene glycol) (PPO-PDMS). The prepared PU-S films showed highly hydrophobic surface properties and decreased absorption of water with the increasing content of the hydrophobic PPO-PDMS segments. The PUs and PU-S coatings with good morphological properties were very useful in biomedical applications, for example, in hard and soft tissue regeneration, scaffolding, and drug delivery systems [77,233,234]. 

PMHS were widely used as ingredients of dental composites and components of cosmetic formulations because they were physiologically nontoxic [235,236]. The hydrolytic stability of silicone–cellulose membranes (in water and in the presence of a cellulase enzyme), which were prepared from (hydroxypropyl) cellulose (HPC) and PMHS, did not increase with the increasing content of PMHS and was rather optimal for certain PMHS: HPC ratio [237].

New coating materials were obtained by grafting poly(ethyleneoxide) (PEO) with different MWs (350, 750, and 2000 g/mol) onto polysilazane (PSZ) via hydrosilylation reaction. The PEO-*graft*-PSZ hybrid coatings were crosslinked with moisture and were able to stop the adhesion of marine bacteria on surfaces. The antiadhesion and antifouling properties of the coatings were tested against three kinds of marine bacteria and were dependent on the chain length of PEO and the grafting density. The copolymer PEO (350 g/mol)-*g*-PSZ with the highest graft density showed the best antifouling activity. The bacterial antiadhesion properties of PEO (2000 g/mol)-*g*-PSZ coatings were more efficient than the PEO (750 g/mol)-*g*-PSZ coatings [238].

Poly(dimethylsiloxane) films were also modified with PHEMA and collagen and formed interpenetrating polymer networks (IPNs). Collagen was grafted through methyl sulfonyl chloride and covalently bound protein layers, as it was documented by means of the ATR-FTIR and XPS methods. The hydrophilicity of the modified samples was improved as a result of the increasing content of PHEMA. The surfaces modified with collagen showed significant cell adhesion and growth as compared with unmodified PDMS samples. The biocompatibility of the PDMS collagen-modified surface was tested with the culture of fibroblast cells (L929) [239].

From composite PDMS matrix membrane and poly(ethylene terephthalate) (PET) reinforced with satin, novel transversal pneumatic artificial muscles were prepared. They showed excellent biocompatibility and competitive static load characteristics as compared to other pneumatic artificial muscles and may find potential applications in the human gastrointestinal tract [240].

## 7. Silicone-Based Drug Delivery Systems

In recent decades, silicone-based drug delivery devices (DDDs) have found growing meaning, e.g., as intravaginal, transdermal, and subdermal methods of the administration of drugs and suitable candidates for the release of hormonal steroids for controlling the estrous cycle. Silicone rubber (SR) is a suitable drug carrier in DDDs. Several kinds of SEs are used for this purpose (high-consistency elastomers, liquid-silicone rubbers, and low-consistency silicones) and other adhesives. Silicone pressure-sensitive adhesives are prepared from linear polysiloxanes and silicone resins. They form a nonpermanent bond with the skin, but also with metals, plastics, or glass. Curing parameters, such as heat and moisture, affect some physicochemical properties of SR and play important roles in the designing and processing of drug delivery systems. Free volume and the high compressibility of silicone compounds were responsible for their permeability of gases and liquids [118,241].

The grafting of different medicines on macromolecules of poly(methylhydrosiloxanes) (PMHS) was described by A. Bachrach and A. Zilkha [242].

Silica nanoparticles are often used for controlled drug delivery owing to their physical and chemical resistance, biocompatibility, versatility, and possibility for functionalization of polysiloxanes [243]. For instance, the nanoporous SiO_2_-CaO-PDMS xerogel materials were applied as delivery systems for metronidazole (MT) [244].

Silicone oils and crosslinked polysiloxanes did not show the harmful effects of subcutaneous, intracutaneous, and intramuscular administrations. The cultures of various tissues of warm-blooded animals are extraordinarily sensitive. They showed no deviation from the usual growth pattern on contact with liquid, semisolid, and rubberlike silicone materials [245]. Silicones were considered biologically and toxicologically inert [246].

Silicone materials, widely used in medical devices, offer versatility and biocompatibility in DDDs, where silicones are incorporated as a matrix, enabling the elution of an active additive or ingredient, e.g., antiviral drugs; antidepressants; antifungals; anxiolytics; nonopioid and opioid analgesics; and vitamins B_6_, D, and E [247,248]. In order to determine the compatibility of silicone with an active drug, its solubility in silicone oil or elastomer was tested [45]. Very important factors are the interactions between drugs, release-enhancing agents, and silicones. In some devices, the drug was incorporated into a silicone matrix core or reservoir, and the outer layer of silicone (not containing drugs) controlled the release on the device [249,250,251]. Silicones of different molecular structures were used for cosmetics, healthcare, and drug delivery (and also in the aerospace industry and photovoltaics). Optimal release rates of drug are 10 to 500 µm per day and are highly dependent on the type of drug and silicone, and also rate-enhancing additives [248].

In the processes of drug or vaccine fabrication, silicone tubing was used [12,252]. Composites of SR and sodium alginate crosslinked with TEOS also served as the drug delivery scaffold. These new silicone–alginate microcapsules formed the matrix for the release of an antidiabetic drug gliclazide [133].

PDMS/silicone resin networks were applied as pressure-sensitive adhesives in different transdermal drug delivery systems [253].

## 8. Physiological Properties of Organosilicon Compounds and Silicones

It is well known that plants, animals, and humans contain silicone nanoparticles. The silicone atoms stimulate the growth of nails and hair. Every day people consume ~0.5 g of silicone with food, but only 20–30 mg is incorporated into the human blood. However, water-soluble oligomers of orthosilicic acids are easily washed from the human body, but a constant concentration of silicone in the blood is controlled by the kidneys. Chloroorganosilanes react with the tissues of the skin and eyes, while tetrasubstituted organosilanes are inert if they do not contain any functional groups. Conversely, amino- and alkoxy-functional silanes are extremely toxic, e.g., (4-aminobutyl)(diethoxy)methylsilane [254]. (Ethoxy)silanes are toxic to the liver and the kidneys [255].

(3-Iodopropyl)silatrane is also toxic (LD50 > 29 mg/kg), and aryl and mercaptomethyl silatranes do not poison bacteria, fungi, and frogs, but they are very toxic to warm-blooded animals [256]. However, methyl, ethyl, ethynyl, chloromethyl, and (1-chloroethyl)silatranes are nontoxic (LD50 > 2000 mg/kg). High toxicity was also detected for organosilyl phosphines, arsines, stibines, bismuthines [257], hexaalkyl stannosiloxanes, and plumbosiloxanes [258].

PDMS finds many technical and medical applications, for instance in cardiac valves, gullets, trachea, face-lifting, and in the construction of artificial limbs. For silicone breast implants, silicone elastomers (SEs) and rubbers (SRs) were used, which were low-MW silicone oils as plasticizers. Catheters, drainage, and tracheotomy tubes were also fabricated from SRs. Synthetic arteries impregnated with silicones were elastic, avoided blood coagulation, and showed good mechanical properties and immunological tolerance. A heart-lung medical equipment for blood circulation was made of SEs and SRs. Low-MW PDMS plasticizers of breast implants are biologically active, but high-MW silicones are physiologically inert. 2,6-*cis*-Diphenylhexamethylcyclotetrasiloxane was used as a drug for decreasing sexual activity and in the treatment of prostate disease [256].

Very small amounts of silicone oils (*dimethicones* or *cyclomethicones*) are components of cosmetics, drugs, and food additives. A low toxicity (LD50 > 15 g/kg) of cyclomethicone vapors was determined (LC50 > 1200 ppm). However, occasional irritations of the epithelial and eyeball and an increase in liver weight in rats that were fed with 5–10% dimethicone or cyclomethicone for 90 days were observed. Alternatively, they were subjected to inhalation of 5% and 10% of dimethicone or cyclomethicone vapors. Studies confirmed crucial problems with the use of breast implants made of SEs, which were plasticized with low-MW PDMS [259]. Low-MW silicone oils penetrate the cell membranes into the blood and undergo partial enzymatic biodegradation. So, greater caution is necessary, especially in the case of their medical applications [259,260,261].

PDMS, used as a food additive (as E-900), should have viscosities in the range 200–300 cSt and MWs ~10,000 g/mol, which correspond to an average degree of polymerization (DP) ~120. Only PDMS with linear structures can be used in pharmaceutical products and in the food industry. Moreover, the daily consumption of PDMS with food should be lower than 1.5 mg/kg of body weight [262,263]. Pharmaceutical products (*Simeticone* or *Dimeticone*) contain PDMS with a DP of 20–400 and a kinematic viscosity of 20–1300 cSt, while low viscous PDMS (<50 cSt) can be used only for external applications [264,265]. The presence of PDMS was found in the liver, lymph nodes, and spleen a few years after the implantation of breast implants [266]. Silicones may affect the conformation change in proteins (e.g., myoglobin) [267]. The biodegradation of silicones in the body was observed in vivo and in vitro [268,269]. After a few months in the blood and liver of animals, many low-MW biodegradable silicone products were detected. The toxicity of the cyclosiloxanes was also evaluated. An intra-peritoneal administration to animals of hexamethylcyclotrisiloxane (D_3_), octamethylcyclotetrasiloxane (D_4_), decamethylcyclopentasiloxane (D_5_), and dodecamethylcyclohexasiloxane (D_6_) led to pneumonia and liver and hepatocellular necrosis [260,261]. The oral administration of food containing D_3_, D_4_, or D_5_ to rats caused hepatomegaly, liver and lung damage in mice [270]. The toxicity of (Me_2_SiO)_4_ (D_4_) was found to be similar to the toxicity of CCl_4_ or trichlorethylene. Cyclosiloxanes migrate from breast implants and accumulate in other body organs: the liver, lungs, blood, and fat, increasing their weight and causing kidney and adrenal hyperplasia in rats [271,272]. Cyclosiloxanes strongly affected the immune response, resulting in acute inflammation, denaturation, and a change in the conformation of the two proteins—fibrinogen and fibronectin. The disease of the immune system was also observed in children nursed by mothers with breast implants. Experimental studies in monkeys [273] and rats [274,275] indicated that the PDMS and cyclosiloxanes (D_n_), after oral administration, were present in the blood and in urine. The negative impact of D_n_ in reproduction and their teratogenic and oncogenic effects were recognized as well [270,276].

Currently, it was confirmed again that low-MW polysiloxanes, especially those that are soluble in water, can penetrate cell membranes and the blood by enzymes that promote their biodegradability [259].

### Antimicrobial Protection of Silicone Implants

**Figure 5:**
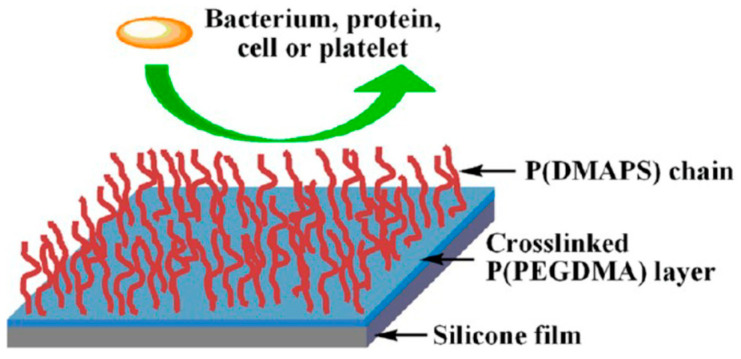
A visualization of a chosen example of the antimicrobial modification of a silicone surface. Reprinted (adapted) with permission from [37]. Copyright (2012) American Chemical Society.

Highly hydrophobic silicone biomaterials are susceptible to bacterial, protein, and biomolecule adhesion, which may cause severe infections even leading to death. In order to avoid it, antibacterial and antifouling properties of medical-grade silicone surface were improved by covalent grafting crosslinked poly(ethylene glycol) dimethacrylate [P(PEGDMA)] (see Figure 5). The P(PEGDMA)-grafted silicone (silicone-*g*-P(PEGDMA)) layer decreased the adhesion of *Staphylococcus aureus*, *Escherichia coli*, *Staphylococcus epidermidis*, and 3T3 fibroblast cells by ≥90%. The enhanced antifouling efficacy and improved hemocompatibility with the preservation of the antibacterial property were observed when polysulfobetaine polymer, poly((2-(methacryloyloxy)ethyl)dimethyl(3-sulfopropyl)ammonium hydroxide), was immobilized on the silicone-*g*-P(PEGDMA) surface, which led to the reduced absorption of bovine serum albumin and bovine plasma fibrinogen by ≥80%. Such antimicrobial effects may potentially improve the long-term properties of silicone-based peritoneal dialysis catheters [37].

Another method for antimicrobial protection on the surface and inside of biomaterial was also elaborated. For this purpose, chemical agents containing silver, free-radical forming agents, antimicrobial peptides, nonsteroidal anti-inflammatory drugs, or quorum-sensing blockers were applied [277]. The covalent bonding of 2-methacryloyloxyethyl phosphorylcholine (MPC) to the interocular silicone lenses (IOLs) increased their hydrophilicity and reduced the surface adhesion of macrophage, lens epithelial cell, and platelet. MPC-modified hydrophilic silicone IOLs showed reduced adherence and colonization of bacteria [1,278]. Similarly, the surface of human silicone breast implants was covalently grafted with an MPC-based polymer. The addition of crosslinkers resulted in the enhanced density and mechanical durability of the MPC graft, which strongly inhibited adsorption and the conformational deformation of fibrinogen, leading to the exposure of a buried amino acid sequence, γ377−395, which was recognized by inflammatory cells [279].

Urinary catheters were sterilized by functionalization, coating, drug impregnation, and blending. Functionalization was conducted with gamma, UV, or plasma irradiation, followed by grafting bioactive agents on the catheter surface which was activated with free radicals. Such modification prevented the adhesion of bacteria. The silicone catheter surface was also impregnated with blends of antimicrobial agents (e.g., chlorhexidine and triclosan) and common antibiotics [280]. Surfaces of silicone peritoneal dialysis catheters were modified with hydrophilic functional polymers, e.g., poly(ethylene glycol) (PEG) [8,281]. A lower bacterial adhesion and 80% bacteria reduction were observed for silicone rubber peritoneal dialysis catheter surfaces modified by dip-coating containing graphene oxide [282].

The silicone urinary catheters coated with biofilms of the extracted chitosan (EC) reduced the adhesion of the pathogens, e.g., slime formation in *S. epidermidis* and destruction of *C. albicans*. Moreover, EC can downregulate the virulence genes in both pathogens [283].

Silicone breast implants coated with Cu(1) and Cu(2) compounds or containing Cu(0) showed antiadhesive and antibacterial properties against *S. epidermidis*. Cu ions were released over time, and copper-coated silicone had the ability to inhibit infections of breast implants [284].

For the antimicrobial protection of breast implants, the following were also applied: incorporation of inorganic oxides (TiO_2_, ZnO, and SiO_2_) and nanoparticles; immobilization of enzymes, peptides, drugs, and bioactive polymers; or adsorption of proteins [98,285,286]. Alternatively, breast implants were immersed in an antiseptic solution (betadine or hydrogen peroxide) before surgery or coated with nanoparticles or antibiotics having antimicrobial properties. The plasma-activated silicone was water-absorbing and formed protective surface coatings with antibiotics (e.g., *Cefazolin* or *Gentamicin*, 10% povidone, and iodine) which inhibited the growth of bacteria [287].

The surface of SE was modified with maleopimaric acid quaternary ammonium cation (MPA-N+) and formed the antimicrobial coating, which was analyzed by EDS, XPS analysis, and SEM. The MPA-N+ film inhibited the growth and multiplication of bacteria (*S. aureus*, *E. coli*, and *P. aeruginosa*). It showed excellent hemocompatibility and no toxic side effects on human aorta smooth muscle cells. The prepared PDMS-g-MPA-N+ may find potential biomedical applications [288].

The antimicrobial properties of silicone implants (SIs) were significantly improved by surface modifications with peptides. Thus, biofilm formation and combat catheter-associated infections were inhibited. Moreover, antimicrobial SI provides many benefits in wound healing [289].

Highly stable lubricant-infused coatings for intranasal splints were prepared by the surface-initiated polycondensation of n-propyl(trichloro)silane followed by infiltrating them with a silicone oil lubricant. Thus, superhydrophobic polysiloxane nanofilament (PSnF) coatings were created. Their surfaces became antithrombogenic and repellent toward bacterial cells. PSnF significantly decreased plasma and blood clot formation and prevented bacterial adhesion and biofilm formation for up to 7 days [290].

## 9. Toxicity and Safety of Siloxanes and Silicones

A global distribution of oligomeric cyclic and linear volatile methylsiloxanes in air was analyzed and described by S. Genualdi et al. [291]. Silicones were generally consi-dered safe. However, according to some studies, silicones are not completely inert and can release small amounts of toxic substances, mostly volatile cyclic dimethylsiloxanes (Me_2_SiO)_n_ (Me = CH_3_): octamethylcyclotetrasiloxane (D_4_), decamethylcyclopentasiloxane (D_5_), and dodecamethylcyclohexasiloxane (D_6_). Cyclic and linear siloxanes contain alternating silicone and oxygen units having organic side chains. According to the European Chemicals Agency, D_n_ may cause serious effects on the environment and human health, and organosiloxanes can cause negative toxic effects on humans and other living organisms. Cyclosiloxanes are ingredients of personal care products, lubricants, and solvents in commercial applications, and in environmental media, they are especially present in sewage sludge. D_5_ is the dominant siloxane in environmental matrices except for air, where D_4_ dominates. Certain siloxanes found in the environment resist oxidation, reduction, and photodegradation. But siloxanes undergo hydrolysis reactions and hydroxylation metabolites may be found in the blood and urine. Human exposure to D_4_ from the use of personal-care products was estimated at ~1 mg per day. Animal toxicity studies of D_4_ found changes in organ weights, induction of hepatic drug-metabolizing enzymes, and adverse effects on reproductive health and function. The weak estrogenic activity of D_4_, in combination with its long half-life, poses potential concerns for exposed individuals. D_4_ exposure was associated with the development of benign uterine tumors (adenomas) in rats. However, the acute LD_50_ of 6–7 g/kg indicated that D_4_ is acutely nontoxic [292,293].

Analyzes of the plasma and blood of women exposed to silicone gel-filled breast implants showed that many years after the removal of ruptured silicone implants, D_4_ and D_6_ were still present in the plasma and in the blood. They were not detectable in the women without implants. In 2008, the total daily exposure to D_5_ from personal care and consumer products in women in the USA was estimated as 233 mg/day. D_5_ caused uterine endometrial adenocarcinomas in female rats and also had adverse health effects on the reproductive system, adipose tissue, bile production, and the immune system through its effects on prolactin, and it has the potential to cause adverse effects on the nervous system because of its influence on the neurotransmitter dopamine. Although studies did not show D_5_ to be estrogenic, it caused uterine tumors in animals. D_6_ was present in select consumer products (range 0.33 to 43.10 μg/g) and daily exposure for women in the USA was estimated at 22.00 μg/day. The liver and thyroid enlargement and reproductive effects were caused by D_6_ exposure. D_6_ has the potential to affect aquatic organisms at concentrations close to their water solubility [292,293].

Low-molecular-weight volatile linear siloxanes: hexamethyldisiloxane (L2), octamethyltrisiloxane (L3), decamethyltetrasiloxane (L4), and dodecamethylpentasiloxane (L5) are generally inert and non-reactive, with low dermal toxicity. They are compatible with a wide range of chemicals and have profitable chemical properties: wash-off or transfer resistance from the skin, sun protection factor (SPF) enhancement, emolliency in cleaning products, etc. Owing to these properties, these compounds are incorporated into multiple consumer products for use on the skin, such as cosmetics and healthcare products, with over 300,000 tons annually sold in the personal care and consumer product sector. Due to their widespread use in consumer products and the potential for human dermal exposure, dermal absorption takes place [294].

Taking into account that siloxanes can accumulate in the human body over time, this could lead to long-term health effects, e.g., carcinogenic and toxic to the nervous, reproductive, and immune systems of animals. According to literature reports, siloxanes can disrupt hormone function, cause developmental and reproductive toxicity, and damage the liver and kidneys. They also show the ability to overcome the barrier of the skin. So far, some silicone additives have already been banned or restricted from use in cosmetics in the EU [295].

The international standard ISO 10993 provides guidelines for evaluating the biological safety of medical devices, including silicone-based components. It includes various tests such as irritation, genotoxicity, and implantation tests in order to rate the compatibility of silicone materials with the human body. ISO 13485 [139] ensures that medical devices, including those made with silicone, are produced under a strict quality management system (Medical Devices Quality Management Systems). This ensures consistency, safety, and reliability in every batch produced.

Medical-grade silicone rubber has high performance, strength, and elongation, and is very resistant to flaws and crack propagation with an excellent fatigue flex life. Toxicological and biocompatibility studies [systemic toxicity, intracutaneous irritation, hemolysis, intramuscular implantation, rabbit pyrogen, *lysosomal acid lipase* (LAL), sterility and safety tests] were carried out to qualify the material and fabricated device as per international regulatory agencies. The results of the authors’ experiment indicated that the material was found to be nontoxic and the device was deemed to pass the mandatory biological tests [296].

### 9.1. Negative Effects of Breast Implants

The migration of silicone particles outside the breast implant capsules was detected. Silicone leakage occurred in 98.8% of women and in 86.6% of women with silicone gel breast implants. The accumulation of silicone in the synovial tissue of the wrist suggested local silicone toxicity [297,298].

The detection of siloxanes and their breakdown products using in vivo tests enabled studies of the toxicity of silicone breast implants, while thanks to the advances in biophysics, syndromes and well-defined rheumatologic disorders were analyzed [299].

As a result of gel leaking and implant rupture, women with silicone gel-filled breast implants are exposed particularly to methylsiloxanes. It was found that they can cause serious health problems. The effects of microdroplets of methylcyclosiloxanes, in parti-cular D_4_, on the viability of cultured human cells were analyzed. During the exposure of Jurkat human T lymphocyte suspension and HeLa monolayer cervical cancer cells to D_4_, morphological changes in the cells were detected. D_4_ and, to a lesser extent, D_5_, can activate cell-death-related pathways in a cell type-specific fashion, and it is suggested that this phenomenon may contribute to the development of Breast Implant Illness (BII) [300].

Silicone gel breast implants (SBIs) consist of two components: an elastomer silicone shell (envelope) and a silicone gel filler (core). It was confirmed that women with SBI suffered from myalgia, arthralgia, myositis or muscle weakness, arthralgia and/or arthritis, chronic fatigue, fever, dry eyes and/or dry mouth (sicca), and cognitive disturbances. The pathogenesis of these adverse effects and the histocompatibility and interaction of silicone (gels and elastomer surfaces) of SBIs with cells and their surrounding tissue (implant–host tissue interface) were the subject of numerous in vitro studies [301,302]. SBI patients also reported many other clinical health complaints—unrefreshing sleep or sleep disturbances, cognitive impairment (concentration problems and memory loss), pyrexia, sicca, and neurological manifestations (e.g., transient ischemic attack and white matter lesions) [301,303]. Moreover, several other symptoms were often observed: headache, alopecia or hair loss, skin abnormalities, gastrointestinal symptoms (irritable bowel syndrome), night sweats and lymphadenopathy, fibromyalgia, Raynaud’s phenomenon, recurrent respiratory tract infections, recurrent cystitis, livedo reticularis, chronic fatigue syndrome, and allergies [301,304]. Autoimmune diseases that occur in SBI patients are rheumatoid arthritis, vasculitis, Sjogren’s syndrome and other connective tissue diseases, granulo- matous disease, and others like multiple sclerosis and Hashimoto’s thyroiditis. SBIs also cause capsular contracture, implant rupture, breast pain, asymmetry, and infection [301,305]. An increased occurrence of a deficient humoral immune system was reported as well [301,306]. In patients with silicone implant incompatibility syndrome, vitamin D deficiency, related to the presence of autoantibodies, was found [301,307]. A long-standing unexplained symptom of direct inflammatory reaction to the distant (sub)cutaneous silicone depositions from ruptured breast implants was observed [308]. ISO 14607:2024 specifies particular requirements for mammary implants [309].

### 9.2. Environmental Effects of Silicone Containing Personal Care and Other Products

Siloxanes can become gaseous when heated. It was found that 4 of 14 silicone baking molds exceeded Germany’s indoor guide level for D_n_, but the health hazard guide level was not exceeded [310]. Moreover, siloxanes can oxidize to formaldehyde during heating to very high temperatures [311].

Most food-grade silicone utensils can withstand very high heat, but heat tolerance for silicone cookware varies. Although silicone rubber from cookware does not react with food or beverages and does not form any hazardous fumes [312,313], it is possible to melt silicone cooking products if they are overheated. This can cause silicone liquid to mix into food. If this happens, the melted product and food should be thrown out. Silicone cookware may not be used at temperatures above 220 °C [314].

Silicones are recyclable but not biodegradable, they do not break down into microplastics either, which makes them ocean-friendly and less harmful than plastic. Silicone products can be recycled, but research studies on the recycling of silicones are in the early stages [315]. However, it was reported that studies of biodegradable silicone are among the most recent inventions. Such a green development should help to dissolve the world problem of this part of medical waste [96]. Silicones are considered eco-friendly, durable, and harmless. A small amount of silicone being used will lead to huge reductions in greenhouse gas emissions. The use of silicone products can reduce CO_2_ emissions by nine times. Products containing silicones show an increased product lifecycle. Silicones disposed of at a landfill for incineration, unlike common plastics, are converted back into inorganic, harmless ingredients: amorphous silica, CO_2_, and water vapor [316].

On the other hand, liquid silicones can be found in rinse-off products; thus, they can pollute the aquatic environment, accumulate in aquatic organisms over time, and potentially cause harm to their health and reproduction. Siloxanes, which are the building blocks of silicones, have been shown to cause toxicity to aquatic microorganisms, including altering their growth, reproduction, and even causing mortality at high concentrations. Furthermore, siloxanes can also impact the food chain as they can be passed from smaller organisms to larger ones, potentially increasing their concentration and toxicity [295].

In the last 15 years, more than 50% of all new cosmetics contained at least one type of silicone component, and it is necessary to monitor the release of these substances into the environment and rate the environmental and ecological risks. Cyclosiloxanes are considered “persistent, bioaccumulative, and toxic” or “very persistent very bioaccumulative” substances. Thus, they undergo new regulations to control their usage in the USA and Canada. In the European Union, they are controlled by the chemical regulation act called REACH (Registration, Evaluation, Authorisation and Restriction of Chemicals). Although the silicone industry claimed that this decision did not find scientific evidence, wash-off cosmetic products containing volatile cyclosiloxanes: D_4_, D_5_, and D_6_ with concentrations ≥ 0.1 wt. % were limited according to the ECHA’s proposal (European Chemicals Agency). Moreover, since May 2019, on the basis of the European Union Cosmetic Regulation 1223/2009, D_4_ may not be added intentionally to cosmetic formulations sold in the European Union. Similar health effects to *cyclomethicones* may cause the presence of linear siloxanes in cosmetics and personal care products. So, further studies of this aspect should be continued [317].

## 10. Conclusions and Future Perspectives

Different kinds of silicones (and especially oils, elastomers, and rubbers) exhibit valuable physical and chemical properties, resistance to cold and high temperatures, and excellent biocompatibility and bio-durability. These features led to a huge number of practical industrial, but also a wide range of numerous medical, cosmetic, and pharmaceutical applications of silicones, their copolymers, and blends, and also uses of silanes and low-molecular-weight siloxanes. They have been described in this quite comprehensive review. Silicones and silane-modified products still have great potential for further development and future uses in these fields.

In recent decades, silicones have been used frequently in the healthcare industry and in drug delivery applications. However, the widespread applications of silicone rubber in medicine such as catheters have some limitations. For instance, they exhibit poor tear strength and poor resistance to fatigue. Brittle fractures can occur from defects within sections due to the poor control of vulcanization. It led to high failure rates for breast implants. It caused a crisis of confidence. Many manufacturers were forced to produce breast implants only under FDA control [129].

The industrial producers of silicones are interested in the effects of their actions on living organisms; thus, they contribute greatly to the development of studies on their toxicity. Although there is quite convincing evidence that siloxanes are relatively nontoxic to the environment, future studies of this problem are necessary to continue [318]. Bio-degradable silicone materials are the subject of the most recent research and inventions. Such a green development should also help to dissolve the world problem of this part of medical waste [96].

It is quite obvious that the future of silicone biomaterials will depend on their antimicrobial properties, especially for such applications as contact lenses, urinary catheters, and breast implants. To fulfill this requirement, the silicone surface coatings should contain (besides functional nanoparticles) effective antibiotics or plasma-activated silicone implants modified with antibiotics in order to inhibit bacterial growth [1].

Owing to excellent biocompatibility, durability, and adaptability, silicones have revolutionized the medical field by providing safe and reliable solutions for diverse healthcare settings. From prosthetics to implants, diagnostic to wearables, its range will bring in improved outcomes for patients, and in general, better healthcare.

The future of silicones in medicine looks to be brighter as it addresses all the modern challenges with a wide range of sustainable solutions based on the latest advancements, for instance, biodegradable silicone, smart devices, and 3D-printed implants [96]. However, the expected further development of the 3D printing of silicone elastomers is still a challenge and should allow their widespread applications in patient-specific medical devices with optimized multifunctional properties in the future [96,141].

## Figures and Tables

**Table 1:** Silanes and silicones used in biomedical applications [13].

1.	Monomers, functional silanes, and low-molecular-weight oil and resins—for surface modifications and molecular coatings, and for curable and crosslinking systems.
2.	Prepolymer fluids, filler-free silicone elastomers, pressure-sensitive adhesives, and gels.
3.	Low- and high-consistency silicone rubbers (RTV, LTV, and HTV systems) most often used as soft tissue substitutes, catheters, flexing joints, drain shunts, and adhesives.
4.	Silicone graft copolymers and interpenetrating polymer networks (IPNs): silicone–urethanes, silicone–polycarbonates, silicone–methacrylates (thermoplastics with improved mechanical properties) useful for cardiovascular devices, contact lenses, and membranes.

**Table 2:** Important fields of healthcare applications of silicones [60].

1.	Orthopedics.
2.	Catheters.
3.	Drains and shunts of numerous descriptions.
4.	Components in kidney dialysis, blood oxygenation, heart bypass machines, and heart valves.

**Table 3:** Major examples of biomedical applications of silicones [60].

1.	Tubing for blood transfer, drugs, or nutrients.
2.	Membranes of blood oxygenators in heart-lung circulation.
3.	Special devices: rings, seals, valves, rods, and sheets.
4.	Implants and encapsulants for plastic, metallic, orthopedic, and other surgical specialties.
5.	Drug delivery systems and aids for pharmaceutical processing.

**Table 4:** Unique properties of SRs [1,81,82,83,84,85,86,87,88,89].

1.	Chemical resistance, excellent oxidation, and UV resistance.
2.	Good elastomeric properties and softness.
3.	High thermal stability and superior aging resistance.
4.	Good resistance to low temperatures.
5.	Sterilizable (e.g., autoclave and gamma irradiation).
6.	Excellent dielectric properties over a wide range of temperatures.
7.	Physiological indifference, satisfactory biocompatibility, FDA food-grade compliance, and perfect biodegradation resistance.

**Table 5:** Most important advantages of silicone rubber [80,89,90].

1.	Heat resistance up to 305 °C and its flexibility even at −100 °C.
2.	Thermal stability: Silicones retain their shape, size, durability, and flexibility in extreme hot or cold temperatures.
3.	High-performing rubber with good mechanical properties and good durability, precision, and reproducibility (i.e., consistency from batch to batch), suitability, and flexibility under extreme heat or cold for a long time.
4.	Environmental resistance against extreme temperatures, UV radiation, corrosive chemicals, and open flames.
5.	Highly versatile SR with the capacity to be molded and shaped in any form providing: flexibility, toughness, and electrical conductivity.
6.	Excellent electrical properties: SRs can be excellent electrical insulators (and their conductive properties can be improved by the addition of metal powders or carbon black).
7.	Moisture sealants: They are the most suitable materials due to their being water-repellent and malleable.
8.	Wide range of hardness: SRs can be formulated to exhibit different flexibility and hardness depending on the kind of application.
9.	Color-friendly: SRs can be molded in any color.

**Table 6:** Representative biomedical applications of SRs.

		Ref.
1.	Medical tubing in heart bypass machines, hoses coated inside with heparin, hydrocephalus, pacemaker leads, shunts for the regulation of cerebro-spinal brain fluid, peristaltic pump tubing, transfer tubing, high-pressure reinforced hoses, gaskets and seals, pacemakers, and stents.	[1,6,13,87,96]
2.	Urinary catheters (including biocidal coatings).	[1,91,95]
3.	Respiratory masks, drains, and shunts.	[1,91,95,96]
4.	Orthopedic parts, prosthetics and orthotics, face prosthetics, and craniofacial prostheses.	[1,87,89,91,95,96]
5.	Silicone restorative finger joint implants and testicular and chin implants.	[1,8,85,96]
6.	Components in kidney dialysis and blood oxygenation, heart bypass machines and heart valves, and artificial heart.	[6,95]
7.	Contact lenses and reconstructive hydrogels.	[1,60,97]
8.	Breast implants and other aesthetic implants widely used in the scrotum, chin, nose, cheek, calf, and buttocks.	[1,3,96,98]
9.	Components of silicone adhesives, gels, and foams as ingredients of various wound dressing compositions.	[1,92,93,94]
10.	Silicone-based bio-scaffolds for cellular therapies.	[97]
11.	Drug delivery systems.	[19,60], see Section 7
12.	Implanted medical or diagnostic devices, non-implant external devices, and components, silicone-based coating of medical apparatus.	[1,89,96]

## Data Availability

No new data were created or analyzed in this study. Data sharing is not applicable to this article.
